# Improved chaos-enhanced FOX for clustering-based supervised medical classification

**DOI:** 10.1038/s41598-026-50872-w

**Published:** 2026-05-06

**Authors:** İlker Dağlı, Onur İnan, Fatih Başçiftçi

**Affiliations:** Department of Computer Engineering, Institute of Science, Selçuk University, Konya, Turkey

**Keywords:** Optimization, Classification, Clustering, Fox optimization algorithm, Chaotic maps, Cancer, Computational biology and bioinformatics, Diseases, Health care, Mathematics and computing, Medical research

## Abstract

Despite the widespread use of optimization-based classification methods in medical data analysis, many existing approaches suffer from premature convergence and limited robustness when dealing with complex and heterogeneous datasets. To address these limitations, this study presents a chaos-enhanced, fox-inspired classification framework derived from the Fox Optimization Algorithm. The proposed method employs a Gauss/Mouse chaotic map to regulate the exploration–exploitation balance through the control variable, while preserving the original algorithmic structure without introducing additional parameters. The framework adopts a clustering-based classification strategy in which cluster centers are optimized using the proposed method, and class labels are assigned via distance-based nearest-neighbor analysis. The approach was evaluated on six publicly available medical datasets, including Breast Cancer Wisconsin Diagnostic, Breast Cancer Wisconsin Original, Dermatology, Thyroid, Hepatitis, and Heart, using accuracy, precision, sensitivity, and specificity as evaluation metrics. Experimental results demonstrate that the proposed framework achieves statistically significant and consistent classification performance, attaining the best overall average rank (1.16) in the Friedman test (p = 0.0012) and outperforming several baseline methods. Performance improvements over benchmark methods were observed across multiple datasets, while comparable results were obtained on others. The incorporation of chaotic dynamics effectively enhances search behavior by mitigating premature convergence. Statistical analyses, including the Friedman test, further confirm the significance of the observed improvements. Overall, the findings indicate that the proposed framework provides stable and reproducible classification performance across benchmark medical datasets. Future studies may extend this work through external clinical validation and alternative methodological integrations.

## Introduction

With the rapid advancement of technology, large volumes of data are continuously generated across various domains. In the medical field, accurate analysis of patient and disease information is essential for effective diagnosis and clinical decision-making. As medical datasets grow in size and complexity, the extraction of reliable and consistent information becomes increasingly difficult. This challenge necessitates the adoption of advanced data processing and analytical techniques. In this context, data mining methods, particularly classification techniques, play a central role by enabling the systematic categorization of medical records based on relevant features. This process supports predictive modeling and informed clinical decision-making^1^.

Optimization techniques are widely employed to improve classification performance by identifying suitable parameter settings and decision boundaries under various constraints^2^. However, classical gradient-based methods often encounter difficulties when applied to nonlinear, high-dimensional, and multimodal medical datasets. For this reason, metaheuristic optimization algorithms have attracted considerable attention due to their flexibility and robustness in complex search spaces, and they have been widely applied in engineering, control systems, decision-making, and forecasting applications^3,4^, with objectives such as cost reduction, risk minimization, and performance improvement^5^. In recent years, chaotic map theory has been increasingly integrated into metaheuristic frameworks to improve the balance between exploration and exploitation, mitigate premature convergence, and enhance solution diversity. In this context, the present study differs from conventional chaos-enhanced metaheuristic approaches. In many existing methods, chaotic maps are incorporated by modifying multiple components of the optimization process, such as position update rules, control parameters, or initialization strategies. In contrast, the proposed CSFOX framework adopts a deliberately minimal and controlled integration strategy in which chaotic dynamics are used exclusively to generate the exploration–exploitation control variable *r*, while all original FOX operators, update equations, and parameters are strictly preserved. This design is not merely incremental but methodological, as it enables an isolated and transparent evaluation of the effect of chaotic regulation on the search dynamics. By preserving the original structure of FOX, the proposed approach minimizes potential confounding factors and allows the observed performance improvements to be primarily attributed to the chaotic control mechanism. This distinguishes CSFOX from prior chaos-enhanced metaheuristic approaches, where multiple algorithmic components are typically modified simultaneously, making it difficult to isolate the specific contribution of chaotic dynamics. Therefore, the contribution of this study lies not in increasing algorithmic complexity, but in establishing a controlled and reproducible framework for analyzing the role of chaos in optimization-driven classification. This controlled design provides a clearer understanding of how chaotic dynamics influence the search process, which is often obscured in more complex hybrid approaches.

Among the various metaheuristic algorithms developed over the past decade are particle swarm optimization (PSO)^6,7^, reptile search algorithm (RSA)^8^, big bang–big crunch (BB-BC)^9^, dynamic arithmetic optimization Algorithm (DAOA)^10^, dynamic water surface walker algorithm (DWSA)^11^, policy optimizer (PO)^12^, bat algorithm (BA)^13^, genetic algorithm (GA)^6^, dwarf mongoose optimization (DMO)^14^, advanced charged system search (ACSS)^15^, symbiotic organism search (SOS)^16^, and hybrid invasive weed optimization–mixed frog leaping algorithm (IWO-SFLA)^17^.

Classification is a central research topic in data mining and aims to learn predictive models that map input attributes to target class labels. These models are subsequently used to classify unseen data, and their effectiveness is evaluated based on the agreement between predicted and actual class labels^1^.

In addition to traditional classification algorithms, optimization-based methods have also been widely adopted. Examples of traditional classification algorithms include k-nearest neighbors (K-NN), decision trees, Naive Bayes, and support vector machines (SVM). Using data mining techniques, existing data can be classified, clustered, or analyzed to uncover relationships, connections, and statistical patterns. This process enables the construction of various predictive models. Once a model is created, it can be used to make predictions on new data that were not included in the original dataset. The accuracy of these predictions reflects the effectiveness of the model in representing the underlying data characteristics. Therefore, determining which algorithm performs best in a data mining application is critical for evaluating the overall success of the application^18^.

Reported classification performances in the literature are obtained under different validation protocols, such as hold-out splits and k-fold cross-validation. These differences may influence reported accuracy levels and limit direct, one-to-one comparisons across studies.

Despite the promising classification performances reported in the literature, several common limitations can be identified across existing approaches. Existing methods often rely on fixed or problem-specific control mechanisms, require extensive parameter tuning, or introduce additional algorithmic complexity to improve performance. Moreover, although chaotic maps have been incorporated into several metaheuristic optimizers, their use in classification-oriented optimization has largely been limited to heuristic integration without a systematic evaluation of robustness, stability, and reproducibility under unified experimental settings.

In particular, there is a lack of clustering-based classification frameworks that exploit chaotic dynamics to regulate the exploration–exploitation balance while preserving algorithmic simplicity and minimizing the number of control parameters. Addressing this gap motivates the development of a chaos-enhanced classification framework that can deliver stable and consistent performance across heterogeneous medical datasets without relying on dataset-specific parameter tuning.

Many methods exhibit sensitivity to parameter settings and dataset characteristics, which may restrict their generalization capability across different medical classification tasks. In addition, fixed or problem-specific control mechanisms adopted by several optimization-based classifiers may lead to premature convergence and insufficient exploration of the solution space. Furthermore, variations in validation protocols and experimental setups complicate reproducibility and hinder fair performance comparisons among studies. These observations highlight the need for robust and transparent optimization-driven classification frameworks that can achieve a balanced exploration–exploitation behavior while ensuring consistent and reproducible evaluation strategies.

Chaos map theory is widely applied in various fields, including solving complex problems, numerical analysis, image encryption techniques, cryptographic applications, and heuristic optimization approaches. The use of chaotic maps helps avoid local optimum trapping and enables the generation of chaotic number sequences that enhance search diversity^19^.

Accurate and early disease diagnosis plays a crucial role in effective treatment and improved patient outcomes. Motivated by this need, optimization-based classification methods have gained increasing attention in medical data analysis. Among the wide range of metaheuristic optimization algorithms proposed in the literature, the fox optimization algorithm (FOX) has recently attracted attention due to its simple structure, limited number of control parameters, and effective exploration–exploitation mechanism inspired by natural hunting behavior. Compared with widely used metaheuristic algorithms such as PSO and HHO, which rely on more complex update mechanisms (e.g., velocity-based updates or adaptive transition strategies), the FOX algorithm provides a more direct and interpretable search structure. This simplicity facilitates the integration of external mechanisms such as chaotic dynamics without introducing additional control parameters or increasing algorithmic complexity. Unlike many population-based algorithms that rely on multiple adaptive coefficients or complex update rules, FOX employs a direct and interpretable transition between exploration and exploitation phases. This structural simplicity makes FOX particularly suitable for integration with chaotic control strategies, allowing search dynamics to be enhanced without increasing algorithmic complexity. Accordingly, FOX provides an effective balance between exploration and exploitation while requiring fewer control parameters, making it a suitable candidate for classification tasks. In the FOX framework, chaotic maps such as the Gauss/Mouse map can be employed to regulate exploration and exploitation phases with balanced probability.

In this study, the FOX algorithm is integrated with chaotic maps and extended into a cluster center–based classification framework, referred to as the chaotic fox classification algorithm (CSFOX), for medical data classification. The novelty of this study does not lie in the generic integration of chaotic maps into the FOX framework, but in the purpose-driven and constrained use of the Gauss/Mouse chaotic map to regulate the exploration–exploitation balance in a clustering-based classification setting through the control variable *r*, while preserving all original FOX update equations and parameters unchanged. The main contributions of this study are summarized as follows.

## Methodological contributions:

- Two classification algorithms are presented: SFOX and CSFOX. SFOX represents the original Fox optimization algorithm adapted for classification, while CSFOX extends this framework by integrating chaotic maps to enhance the exploration–exploitation balance.
- The integration of chaotic dynamics in CSFOX reduces the likelihood of premature convergence and local optimum trapping by improving the regulation of stochastic search behavior, while preserving the original FOX structure.
- A clustering-based classification strategy is employed, in which optimized cluster centers are obtained using SFOX and CSFOX, and final class labels are determined through distance-based nearest-neighbor analysis, enabling a direct and fair comparison between the two algorithms.

## Experimental contributions:

- Extensive experiments are conducted on six publicly available medical datasets obtained from the UCI Machine Learning Repository and other public sources, including BCWD, BCWO, Dermatology, Thyroid, Hepatitis, and Heart.
- Experimental results demonstrate that CSFOX performs competitively and, in several benchmark cases, favorably compared to the baseline SFOX algorithm and other benchmark methods under identical experimental conditions.
- The findings indicate that integrating chaotic maps into the FOX framework enhances classification performance in benchmark medical datasets, highlighting the potential of CSFOX as a stable and consistent classification framework under controlled experimental settings and as a foundation for future research toward clinical decision support applications.

The UCI database^20^ has emerged as a prominent resource and is frequently referenced in studies such as^21–24^ due to its inclusion of numerous benchmark datasets and its widespread acceptance among researchers. In this study, the effectiveness of the proposed algorithm was evaluated on six medical datasets obtained from the UCI Machine Learning Repository and other publicly available sources, including Dermatology, Hepatitis, Thyroid, BCWD, BCWO, and Heart. The classification performance was comparatively evaluated against existing studies in the literature that employed the same benchmark datasets. The remainder of the paper is structured as follows. Section "Related works" presents the related works. Section "Methods" describes the classification problem, the FOX algorithm, chaotic map methods, and the evaluation criteria used. Section "Chaotic fox classification algorithm (CSFOX)" details the proposed CSFOX method. Section "Classification with the chaotic fox classification algorithm (CSFOX)" presents the classification application. Section "Experimental results and analysis" reports the experimental results obtained by the CSFOX method. Section "Discussion" provides a comparative discussion of the proposed CSFOX approach with other algorithms reported in the literature. Finally, Sect. "Conclusion" concludes the study and outlines future research directions.

## Related works

This section reviews existing studies on medical data classification and optimization-based learning approaches. In particular, it summarizes traditional classification techniques, feature selection strategies, and metaheuristic-driven frameworks that have been applied to medical diagnosis and decision support systems.

Recent advances in data mining, machine learning, and optimization techniques have enabled the development of effective medical decision support systems. Consequently, a wide range of classification algorithms, feature selection methods, and optimization-driven learning frameworks have been investigated in the literature for medical diagnosis and prognosis tasks.

Al-Kahlout and colleagues employed the JustNN model, a neural network–based backpropagation feedforward methodology, to classify Erythematosquamous Diseases (ESD) with high accuracy. Their experimental results showed that the proposed model achieved an accuracy rate of 98.36%^25^. Hameed and colleagues proposed an adaptable learning model for the traditional self-organizing map (SOM) algorithm to rapidly identify optimal weights. The experimental results demonstrated that their approach was more effective than the other algorithms included in the comparison^26^. Liu and colleagues applied the C4.5 classification tree method to medical diagnosis and incorporated RELIEFF for feature weighting. Their findings indicated that this approach improved the precision of medical data classification and enhanced its effectiveness as a decision support tool for clinicians^27^. Huang and colleagues investigated classification accuracy for multiclass problems and reported that their proposed method, which combined feature selection with SVM-RFE techniques, achieved an accuracy rate exceeding 95%^28^. Peng and colleagues presented an explainable machine learning framework that integrates advanced models and techniques to predict the risk of hepatitis disease flare-ups. Their experimental outcomes showed that the random forest (RF) algorithm achieved the highest overall accuracy rate of 91.9%^29^. Novichasari and colleagues combined various classification methods, including support vector machines (SVM), Naive Bayes, C4.5, and neural networks, with the particle swarm optimizer for heart disease diagnosis. This integration resulted in improved classification accuracy^30^. Joshi and colleagues examined several data mining classification methods used in healthcare decision-making systems across different datasets. Their findings indicated that identifying the most suitable algorithm for medical decision support systems is a complex and nontrivial task^31^. Mitra and colleagues conducted experiments using the Incremental Backpropagation Network (IBPLN) and Levenberg–Marquardt (LM) algorithms with a reduced feature set obtained through rough set–based feature selection^32^. Tan and colleagues proposed a hybrid method that combines support vector machines (SVM) with the genetic algorithm (GA) for feature selection within an iterative framework^33^. Shivastuti and colleagues evaluated random forest (RF) and support vector machines (SVM) for diagnosing thyroid disorders and reported that SVM outperformed RF based on the adopted performance metrics^34^. Sivasakthivel and colleagues compared different classification methods, including J48, Random Forest (RF), and classification and regression trees (CART), for thyroid disease prediction^35^. Doğantekin and colleagues proposed a Generalized Discriminant Analysis and wavelet-supported support vector machine (GDA–WSVM) system for thyroid disorder diagnosis. Their method achieved a classification accuracy of 91.86%^36^. Polat and colleagues developed a hybrid approach that integrates the artificial immune recognition system (ARIS) with fuzzy-weighted preprocessing for thyroid disease diagnosis. Using a tenfold cross-validation strategy, their experiments resulted in an accuracy of 85%^37^. Assegie proposed an optimized k-nearest neighbors (KNN) classification model in which optimal parameter values were identified through grid search. The experimental results demonstrated that the tuned KNN model outperformed the version using default parameters for breast cancer detection^38^. Yang and a colleague conducted a comparative study of the Relief and ReliefF algorithms using various datasets^39^. Abdel-Baset and colleagues developed a feature selection and classification framework based on the grey wolf optimizer (GWO) integrated with the k-nearest neighbors (KNN) classifier. They evaluated the effectiveness of this approach through classification experiments on multiple datasets^40^. Lavanya and a colleague examined the impact of feature selection on the classification performance of the decision tree classifier (CART)^41^. Jijitha and a colleague investigated breast cancer diagnosis using k-nearest neighbors (KNN) and logistic regression (LR) classifiers after applying genetic algorithm–based feature selection^42^. Bayrak and colleagues compared support vector machines (SVM) and artificial neural networks (ANN) on the Wisconsin Breast Cancer (Original) dataset using performance metrics such as ROC area, recall, precision, and accuracy. Their results indicated that SVM achieved the highest classification accuracy^43^. Janghorbani and colleagues proposed a hybrid framework known as the Fuzzy Evidence Network to address epistemic uncertainty in medical prognosis and diagnosis. Their experimental findings demonstrated notable improvements in prognostic and diagnostic performance compared with several machine learning algorithms^44^. Das and a colleague applied principal component analysis (PCA) for feature extraction and employed the particle swarm optimizer (PSO) to tune support vector machines (SVM) using linear, polynomial, and radial basis function (RBF) kernels. Their study reported improved classification accuracy across multiple datasets^45^. Abonyi and a colleague proposed a fuzzy model structure for classical fuzzy classifiers in which each rule can represent multiple classes with varying membership probabilities. They evaluated the proposed structure using two different datasets^46^. Khourdifi and Bahaj applied a hybrid approach that integrates the particle swarm optimizer with the ant colony optimizer for heart disease classification. Their results demonstrated that this optimization-based framework outperformed conventional classification methods^47^.

The existing literature on optimization-based medical data classification can be broadly categorized into two complementary research streams. The first stream includes classical and non-chaotic learning and optimization approaches, such as traditional classifiers, feature selection strategies, and metaheuristic-driven frameworks without explicit chaotic control. The second stream focuses on chaos-enhanced metaheuristic methods in which chaotic dynamics are incorporated to regulate exploration–exploitation behavior and improve optimization-driven classification performance.

Recent studies have shown that integrating chaotic mechanisms into metaheuristic optimization algorithms can significantly enhance search efficiency and robustness in classification-oriented learning tasks. In medical data analysis, a chaotic binary ant lion optimizer has been proposed for wrapper-based feature selection on medical datasets, including COVID-19 case studies, and has demonstrated improved classification accuracy in high-dimensional clinical data^48^. Similarly, chaos-enhanced firefly-based frameworks have been applied to real-world classification problems such as COVID-19 fake news detection, showing that chaotic dynamics can enhance population diversity and prevent premature convergence^49^. Chaotic firefly algorithms have also been used to optimize dropout regularization parameters, highlighting the potential of chaos-driven search strategies to improve generalization-oriented optimization processes^50^. In addition, chaotic Harris hawks optimization combined with quasi-reflection-based learning has been employed to enhance convolutional neural network design for medical image classification, suggesting that chaotic mechanisms can contribute to both feature-level and architecture-level optimization^51^. Moreover, a chaotic oppositional whale optimization algorithm integrated with firefly search has been reported for medical diagnostic applications involving simultaneous feature selection and classifier tuning^52^. Collectively, these studies demonstrate that chaos-enhanced metaheuristic frameworks represent a reliable and increasingly adopted strategy for improving optimization-driven classification performance. These findings provide a strong methodological foundation for the chaos-enhanced FOX-based classification framework proposed in this study. Despite these promising developments, several important limitations can be identified in the existing literature. Many chaos-enhanced metaheuristic approaches improve performance by modifying multiple components of the optimization process, such as initialization strategies, control parameters, or position update mechanisms. While such modifications may enhance performance, they also increase algorithmic complexity and make it difficult to isolate the specific contribution of chaotic dynamics.Furthermore, most existing studies focus primarily on performance improvement without providing a systematic evaluation of stability, robustness, and reproducibility under unified experimental conditions. In addition, clustering-based classification frameworks that integrate chaotic dynamics in a simple, controlled, and interpretable manner remain limited in the literature. These observations reveal a clear research gap, which motivates the development of the proposed CSFOX framework, designed to provide a controlled, low-complexity, and reproducible chaos-integrated classification approach.

## Methods

### Classification

Classification is a supervised learning process that aims to assign data samples to predefined class labels based on their feature representations^53^. In a typical classification framework, a model is trained using labeled data to capture the relationship between input features and class memberships. The learned model is subsequently used to predict the class labels of unseen samples. The performance of a classification model is evaluated by comparing predicted labels with ground-truth class values in the evaluation dataset using standard performance metrics. In this study, the classification task is considered within an optimization-driven framework. Within this framework, the learning process is guided by the identification of representative structures in the feature space.

### Fox optimizer (FOX)

The Fox Optimization Algorithm (FOX) was introduced by Mohammed and Rashid (2023) as a nature-inspired metaheuristic based on the hunting behavior of red foxes in snowy habitats^54^. Red foxes maintain effective performance under harsh environmental conditions, which is adopted as an analogy for robustness in search processes^55^. In natural settings, prey localization is achieved by interpreting time differences in ultrasonic signals, followed by directed jumps toward the estimated target location^56,57^.

**Fig. 1:**
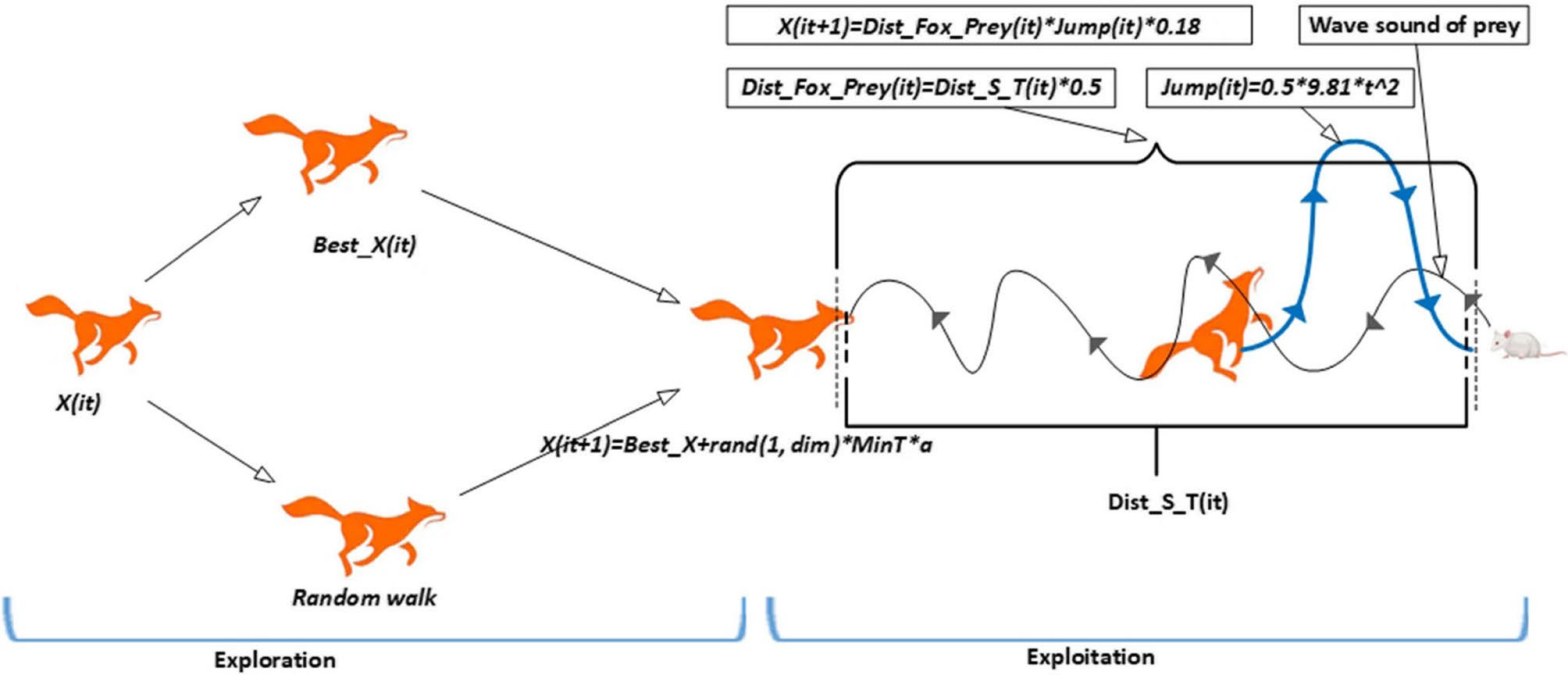
The predatory tactics of the red foxes.

In FOX, this biological behavior is modeled as a population-based search mechanism that alternates between exploration and exploitation phases. The exploration phase promotes stochastic search across the solution space to maintain diversity, while the exploitation phase focuses on refining promising candidate solutions. This balance supports convergence while reducing the risk of premature stagnation. The underlying biological inspiration is illustrated in Fig. 1, which presents the ultrasonic distance estimation principle and its abstraction into a distance-driven search mechanism.

At the beginning of the FOX algorithm, a population of candidate solutions is initialized and stored in a position matrix *F*, where each row represents a fox in the search space. In each iteration, candidate solutions are evaluated using the objective (fitness) function, and the best fitness value (*BestEf*) along with its corresponding solution (*BestF*) is identified. This evaluation is repeated across iterations, allowing the algorithm to retain improved solutions.

To balance global exploration and local exploitation, FOX employs a stochastic control mechanism that determines the search phase with approximately equal probability. This mechanism dynamically guides the algorithm to either explore new regions or refine promising solutions, thereby reducing the risk of premature convergence. As iterations progress, the control parameter *a* gradually decreases, increasing the tendency toward exploitation and improving convergence accuracy. If a position update does not yield improvement, alternative strategies are applied to maintain search diversity and avoid stagnation in local optima.

#### The process of exploitation

During the exploitation phase, FOX performs localized search by guiding each agent toward promising regions identified in previous iterations. This behavior is controlled by a stochastic parameter *p* ∈ [0,1], which determines whether the agent intensifies the search around its estimated target location. When *p* exceeds the predefined threshold of 0.18, inherited directly from the original FOX algorithm^54^, the agent updates its position by estimating the distance to the prey and moving toward that location. To preserve the original algorithmic structure and isolate the effect of the proposed chaotic enhancement, all FOX-specific control parameters were kept unchanged.

To model this behavior, the algorithm computes the traveled sound distance *Dst_S_T* based on the sound travel time *Tm_S_T* ∈ [0,1] and the sound speed *SpS*. In Eqs. (1), (2), (3), (4), (5), (6), (7), (8) and (9), *r* ∈ (0,1) denotes the control variable that governs the selection between exploration and exploitation, while *p* ∈ (0,1) regulates alternative exploitation strategies. *BestF* and *BestEf* represent the current best solution and its fitness value, respectively. *Tm_S_T* denotes the normalized sound travel time, *SpS* represents the sound speed, *Dst_S_T* is the traveled sound distance, and *Dst_F_Prey* is the estimated fox–prey distance. The parameters *kk* and *Min_T* are intermediate transition-time variables, Jmp denotes the leap magnitude, and *c1* and *c2* are fixed directional coefficients inherited from the original FOX algorithm. Accordingly, the traveled sound distance is defined as follows^58^.

1 $$ {\mathrm{Dst\_S\_T}}_{{}} {\text{ = SpS*Tm\_S\_T}}_{{}} $$

After defining the traveled sound distance in Eq. (1), the sound travel time *Tm_S_T* is modeled as a random scalar within the interval [0,1], representing the normalized propagation duration between the prey and the fox. In the FOX framework, the sound speed *SpS* is adaptively estimated based on the current search dynamics, using the best solution (*BestPos*) and the corresponding travel time *Tm_S_T*^54,59,60^. This formulation allows *SpS* to be dynamically adjusted according to the evolving search state. Accordingly, the adaptive sound speed is defined as follows:

2 $$ {\text{SpS = }}\frac{{{\mathrm{BestPos}}_{{}} }}{{{\mathrm{Tm\_S\_T}}_{{}} }} $$

**Fig. 2:**
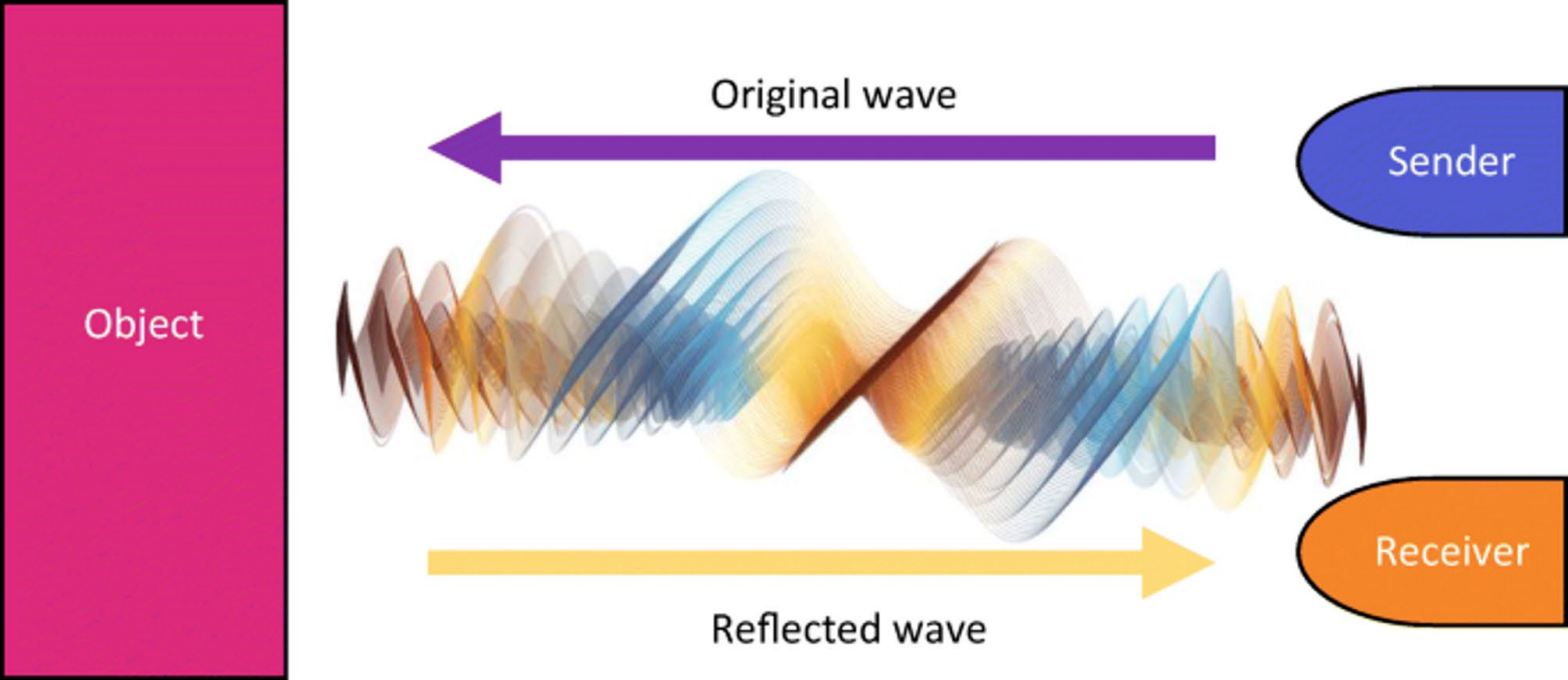
Distance measurement with ultrasonic sensors ^58^.

Once the traveled sound distance *Dst_S_T* is obtained, it is used to estimate the distance between the fox and the prey, denoted as *Dst_F_Prey*. Since the measured sound distance corresponds to a round-trip propagation, the actual separation is computed as half of this value^58^. Figure 2 illustrates this ultrasonic ranging principle. Accordingly, the fox–prey distance is defined as follows:

3 $$ {\mathrm{Dst\_F\_Prey}}_{{}} {\text{ = Dst\_S\_T}}_{{}} {*0}{\mathrm{.5}} $$

Figure 2 illustrates how the round-trip sound distance is converted into the actual fox–prey separation, forming the basis of the distance-driven movement in FOX. After estimating this distance, the algorithm computes the leap magnitude *Jmp*, which represents the movement toward the target during the exploitation phase. The leap is modeled using kinematic principles based on the average transition time *k*, accounting for both upward and downward motion. Accordingly, the leap magnitude is computed as follows:

4 $${\text{Jmp = 0}}{.5*9}{\mathrm{.81*k}}^{{2}}$$

In this formulation, the gravitational acceleration is taken as 9.81, and k denotes the mean transition time derived from the sound travel process. It is computed from the intermediate parameter *kk*, which represents the average sound travel time across all dimensions, obtained by dividing *Tm_S_T* by the number of dimensions. The mean transition time *k* is then defined as *kk/2*, reflecting the bidirectional nature of the motion. The detailed computation of kk and the minimum transition time *Min_T* is provided in Eq. (7).

Following the computation of the leap magnitude, the fox updates its position by combining the estimated distance to the prey with the leap dynamics. This update is regulated by the directional control parameter *c1* ∈ [0, 0.18], as defined in the original FOX formulation. When the stochastic parameter *p* exceeds 0.18, the position is updated as follows:

5 $$ F_{{\left( {\text{it + 1}} \right)}} {\text{ = Dst\_F\_Prey}}_{{}} {\mathrm{*Jmp}}_{{}} {*}\;\:{\mathrm{c}}_{{1}} $$

During the exploitation phase, FOX employs two alternative position update rules that differ in the scaling of the leap magnitude. The selection between these rules is governed by the stochastic parameter *p* ∈ [0,1]. When *p* exceeds the predefined threshold, the position is updated using Eq. (5) with scaling factor *c1*; otherwise, Eq. (6) is applied with scaling factor *c2*^54,59,60^. These mechanisms collectively regulate the direction and intensity of the movement during exploitation.

6 $$ F_{{\left( {\text{it + 1}} \right)}} {\text{ = Dst\_Fx\_Prey}}_{{}} {\mathrm{*Jmp}}_{{}} {\mathrm{*c}}_{{2}} $$

The parameters *c1* and *c2* are directional control coefficients that regulate the intensity and orientation of movement. In accordance with the original FOX algorithm, *c1* ∈ [0, 0.18] and *c2* ∈ [0.19, 1], and in this study they are fixed as *c1* = 0.18 and *c*2 = 0.82. The selection between *c1* and *c2* determines the degree of exploitation or diversification in the position update. This mechanism maintains a balance between intensification and controlled exploration, reducing the risk of premature convergence.

#### Exploration process

During the exploration phase, FOX performs a stochastic search to explore previously unvisited regions of the solution space. Unlike exploitation, this phase relies on randomized movements rather than directed jumps, where the search is guided by the current best solution *BestF*. The exploratory behavior is regulated by two control variables*, Min_T* and *a*, which influence the step size and direction of the random walk.

The parameter *Min_T* represents the minimum transition time and is derived from the intermediate variable *kk*, which denotes the average sound travel time across all dimensions. Specifically, *kk* is computed from *Tm_S_T*, and the minimum value among all *kk* values is selected to obtain *Min_T*, as defined in Eq. (7)^54,59,60^.

7 $$ {\text{kk = }}\frac{{{\mathrm{sum}}\left( {{\mathrm{Time}}_{{{\mathrm{S}}_{{\mathrm{T}}} }} { }\left( {\mathrm{j,:}} \right)} \right)}}{{{\mathrm{dim}}}}{,}\quad {\text{Min\_T = min(kk)}} $$

Following the computation of *Min_T*, the parameter a is updated as a function of the maximum number of iterations (*Maxit*), as defined in Eq. (8). As iterations progress, a gradually decreases, reducing the exploration step size and enabling a smooth transition from global exploration to local exploitation.

8 $${\text{a = 2*}}\left( {{1} - \left( {\frac{{1}}{{{\mathrm{Max}}_{{{\mathrm{it}}}} }}} \right)} \right)$$

To introduce stochasticity, a random vector (*rand(1, dim)*) is used to perturb the fox’s movement, while a scalar parameter r regulates the balance between exploration and exploitation. The new position is then updated according to Eq. (9).

9 $$ F_{{\left( {\text{it + 1}} \right)}} {\text{ = BestF}}_{{}} {\mathrm{*rand}}\left( {\mathrm{1,dim}} \right){\mathrm{*Min\_T*a}} $$

**Fig. 3:**
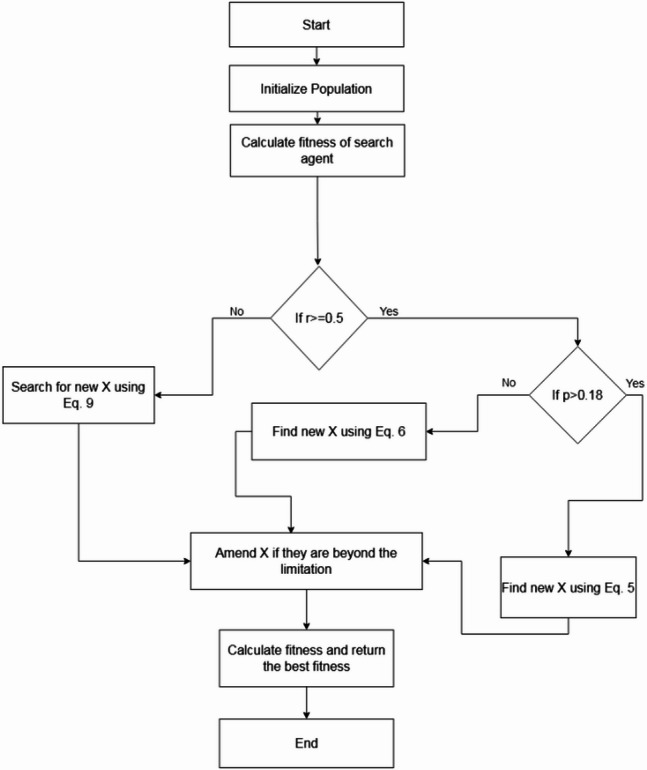
Flowchart of the FOX Algorithm.

**Fig. 4:**
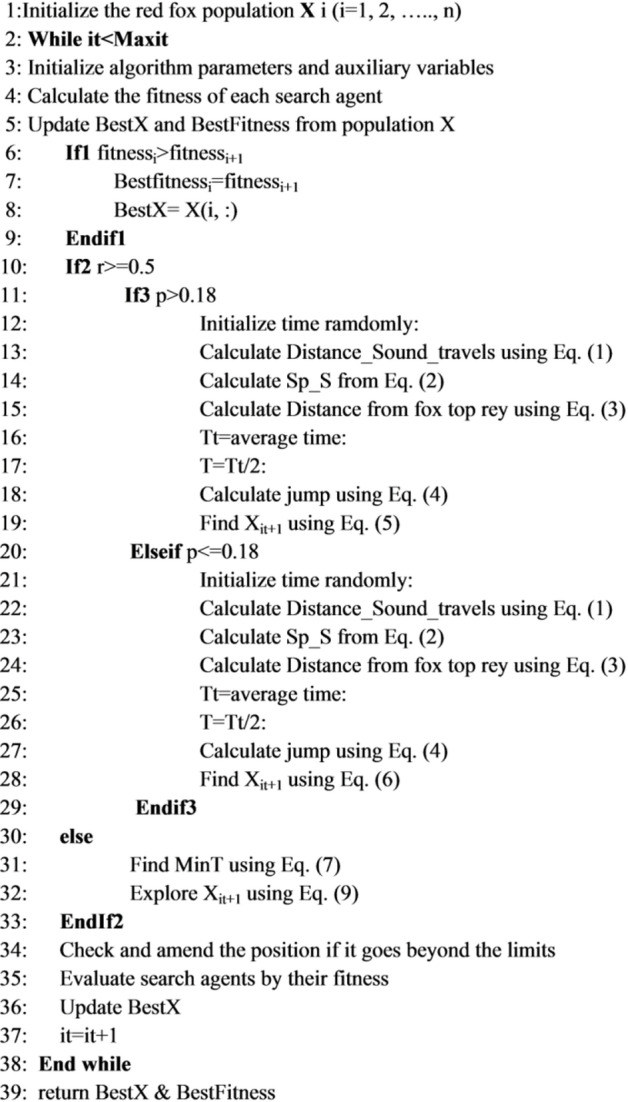
Pseudo code of the FOX algorithm.

This update rule enables effective exploration of the search space while reducing the risk of local optima and premature convergence. The equations used in both exploration and exploitation phases are directly inherited from the original FOX algorithm without structural modification. The overall workflow of the FOX algorithm is illustrated in Fig. 3, and the corresponding pseudocode is presented in Fig. 4.

Figures 3 and 4 illustrate the workflow and pseudocode of the FOX algorithm. Figure 3 provides an overview of the interaction between exploration and exploitation phases, while Fig. 4 presents the step-by-step implementation.

The FOX algorithm initializes a population of candidate solutions within the search space and evaluates them using the objective function. The best solution (*BestF*) and its fitness (*BestEf*) are identified and updated iteratively. At each iteration, a stochastic parameter *r* determines whether the algorithm performs exploitation or exploration. During exploitation, position updates follow the rules in Sect. "The process of exploitation", where the parameter *p* selects between the update formulations in Eqs. (1), (2), (3), (4), (5) and (6). During exploration, positions are updated using a stochastic random walk guided by the current best solution, as defined in Eq. (9). This process continues until the maximum number of iterations is reached, yielding the final solution.

### Chaotic maps

In many heuristic methods, the use of long-period random number sequences plays a significant role. The generated numbers should be non-repetitive, well distributed over a wide range, computationally efficient to produce, and require minimal storage cost. Accumulation within a restricted range or repetition of identical values in randomly generated sequences can increase the likelihood of an algorithm becoming trapped in local minima^19^. The application of chaotic maps can effectively mitigate this limitation by reducing the risk of premature convergence and local optimum entrapment. Chaotic number sequences have been successfully applied in various fields, including artificial neural networks^61^, chaotic map–integrated particle swarm optimization^62^, chaotic brainstorming optimization^63^, and multi-objective transformer design using chaotic evolutionary approaches^64^. Generating and storing chaotic sequences is computationally simple and efficient, as it requires only a basic nonlinear function and a small set of initial parameters to produce sequences of arbitrary length^65^. From a theoretical perspective, it has been shown that numbers generated by chaotic systems exhibit properties such as randomness, ergodicity, broad spectral characteristics, and non-periodicity^66^. The proposed algorithm is based on a conditional control expression that plays a critical role in regulating the balance between exploration and exploitation while avoiding local optima. To achieve an approximately equal probability between the search and refinement phases of the algorithm, the value of the random variable used in the conditional expression is generated through a chaotic mapping mechanism rather than a conventional uniform random generator. In this study, the Gauss/Mouse chaotic map is employed to generate the control variable *r*, which dynamically balances the search and refinement phases during the application of the Fox Optimization Algorithm to the classification process. These theoretical properties of chaotic systems provide a conceptual basis for their use in optimization. In particular, ergodicity ensures a more comprehensive coverage of the search space, while non-periodicity and sensitivity to initial conditions introduce diversified search trajectories. In the context of the proposed CSFOX framework, these properties directly support the regulation of the exploration–exploitation balance through the control variable r, enabling a more stable and effective search process without modifying the original FOX structure.

#### Gauss/mouse chaotic map

In Eq. (10), *x*_*r*_ denotes the current chaotic state, while *x*_*r+1*_ represents the subsequent state generated by the Gauss/Mouse chaotic map. The chaotic sequence produced by this mapping yields values within the interval (0,1). In the CSFOX framework, these values are directly assigned to the control variable *r*, which is used to regulate the probabilistic switching between the exploration and exploitation phases of the FOX algorithm.

10 $$x_{r + 1}^{{}} = \left\{ {\begin{array}{*{20}c} {0,} & {x_{r} = 0} \\ {1/X_{r} \bmod (1),} & {X_{r} \in (0,1)} \\ \end{array} 1/X_{r} \bmod (1) = \frac{1}{{X_{r} }} - \left[ {\frac{1}{{X_{r} }}} \right]} \right.$$

### Dataset

Table 1 presents the datasets and their characteristics. These datasets were obtained from the UCI Machine Learning Repository and other publicly available sources and are commonly used as benchmark datasets in the literature.

**Table 1:** Attributes of the datasets.

Dataset	Class count	Attribute count	Sample count
Dermatology	6	34	366
BCWD	2	30	569
BCWO	2	10	683
Thyroid	3	5	215
Hepatitis	2	19	155
Heart	2	14	1888

Prior to classification, all datasets were subjected to a standardized preprocessing stage to ensure consistency and reliability. Datasets containing missing values were handled using a unified preprocessing strategy across all experiments. Specifically, numerical missing values were imputed using a mean-based imputation approach, which preserves the dataset size and avoids information loss that may arise from record elimination. This preprocessing procedure was applied consistently across all datasets to prevent dataset-specific bias and to ensure fair, transparent, and reproducible experimental comparisons. A detailed description of the datasets is provided below.

- *Dermatology*: The dataset contains 366 records and 34 features, including 33 integer-valued clinical attributes and one nominal attribute. Except for family history and patient age, all clinical features take integer values ranging from 0 to 3, reflecting their presence and severity. Family history is encoded as 1 when relevant conditions are present in the patient’s relatives and as 0 otherwise.
- *BCWD*: The Breast Cancer Wisconsin Diagnostic Dataset (WDBC) consists of 569 samples, of which 357 are labeled as benign and 212 as malignant. Each sample includes 30 descriptive numerical features derived from imaging-based measurements of breast masses, along with one diagnostic class label.
- *BCWO*: The Breast Cancer Wisconsin Original dataset differs from BCWD and contains 683 samples after removing records with missing values. The dataset includes 10 features, with class values ranging from 1 (least abnormal) to 10 (most abnormal), and consists of approximately 65.5% benign and 34.5% malignant instances.
- *Thyroid*: The dataset contains 215 samples categorized into three classes: hyperfunction, hypofunction, and normal thyroid activity. It includes five biochemical attributes related to thyroid function, such as serum thyroxine, triiodothyronine (T3), TSH levels, and response to TRH stimulation.
- *Hepatitis*: The dataset consists of 155 samples with two class labels representing survivor and non-survivor cases. It includes 19 attributes comprising both integer- and real-valued clinical measurements related to hepatitis diagnosis and patient condition.
- *Heart*: Heart dataset consists of 1,888 samples merged from five publicly available heart disease datasets. It includes 14 attributes representing medical and demographic factors related to cardiovascular risk assessment and is labeled for binary classification.

### Evaluation criteria

Various evaluation criteria and metrics are used to assess the performance of algorithms. Evaluating the effectiveness of classification methods is essential for measuring classifier performance. Validation methods are commonly employed to assess classification accuracy and to determine how accurately predictions match the true class labels. Correctly classified instances are those for which the predicted labels coincide with the actual class labels, whereas incorrect classifications are treated as errors. Another widely used measure of classifier effectiveness is the error rate, together with the proportion of correctly predicted class labels in the dataset. In this study, classification accuracy is adopted as a primary performance metric and is defined in Eq. (11). The variables used in Eq. (11) are defined as follows: *TrPos* and *TrNeg* denote the numbers of correctly classified positive and negative samples, respectively, while *FlsPos* and *FlsNeg* represent the numbers of incorrectly classified positive and negative samples.

11 $$Acc = \frac{TrPos + TrNeg}{{TrPos + FlsPos + TrNeg + FlsNeg}}\,\,$$

12 $$Sens = \frac{TrPos}{{TrPos + FlsNeg}}\,\,$$

13 $$Spec = \frac{TrNeg}{{TrNeg + FlsPos}}\,\,$$

14 $$F1 = \frac{2*\Pr ecision*Sensitivity}{{\Pr ecision + Sensitivity}}\,\,$$

Precision denotes the proportion of correctly predicted positive samples among all samples predicted as positive. *TrPos* represents the number of correctly classified samples belonging to the positive class, while *TrNeg* denotes the number of correctly classified samples belonging to the negative class. *FlsPos* indicates the number of samples that belong to the negative class but are incorrectly classified as positive, whereas *FlsNeg* represents the number of samples that belong to the positive class but are incorrectly classified as negative. Sensitivity (Sens) measures the ability of a classifier to correctly identify true positive cases, while specificity (Spec) reflects the ability of the classifier to correctly identify true negative cases. In this study, classification accuracy is primarily used for comparative evaluation with existing studies in the literature. In addition, sensitivity, specificity, and F1-score are reported to provide a more comprehensive and balanced assessment of classification performance.

## Chaotic fox classification algorithm (CSFOX)

The Chaotic Fox Classification Algorithm (CSFOX) is developed by integrating the original Fox Optimization Algorithm (FOX) with chaotic dynamics to enhance search robustness and classification performance. As discussed in Sect. "Chaotic maps", chaotic maps exhibit desirable properties such as ergodicity, non-periodicity, and sensitivity to initial conditions, which are particularly effective for regulating stochastic search processes in metaheuristic optimization. In classification-oriented optimization, maintaining an effective balance between exploration and exploitation is crucial to avoid premature convergence and to improve solution quality. To address this requirement, CSFOX employs chaotic sequences to generate the stochastic control variable *r*, which governs the switching between global exploration and local exploitation within the FOX framework. In CSFOX, the Gauss/Mouse chaotic map is used exclusively to generate the exploration–exploitation control variable *r*, without modifying any FOX position update rules or introducing additional control parameters.

Several chaotic maps were initially examined, including Sine, Logistic, Tent, Chebyshev, Singer, Gauss, Circle, Piecewise, Sinusoidal, Iterative, and Gauss/Mouse maps. Based on the general characteristics of chaotic systems outlined in Section "Chaotic maps" and their reported behavior in optimization contexts, the Gauss/Mouse chaotic map was selected for use in CSFOX. To quantitatively support this selection, an ablation analysis was conducted by integrating multiple chaotic maps namely Chebyshev, Circle, Iterative, Logistic, Piecewise, Sine, Singer, Sinusoidal, Tent, and Gauss/Mouse into the CSFOX framework.

All chaotic variants were evaluated under identical experimental conditions across six medical datasets. For each configuration, the mean classification accuracy, standard deviation, and rank-based performance metrics were computed as the average of 30 independent runs to ensure statistical reliability. The comparative results, summarized in Table 2, indicate that although several chaotic maps achieve competitive performance on individual datasets, the Gauss/Mouse chaotic map consistently demonstrates superior overall behavior by attaining the lowest average and final ranks. Moreover, its lower standard deviation values indicate improved stability and reduced sensitivity to stochastic fluctuations.

**Table 2:** Quantitative ablation analysis of chaotic map selection across medical datasets.

Dataset	M	Chebys	CSFOX	Circle	Iterative	Logistic	Piecew	Sine	Singer	Sinusoidal	Tent
BCWD	A	%96.02	**%98.15**	%95.99	%96.05	%95.76	%95.67	%96.31	%96.14	%96.34	%96.19
	S	0.014	**0.0053**	0.017	0.019	0.017	0.023	0.018	0.018	0.015	0.016
	R	7	**1**	8	6	10	9	3	5	2	4
BCWO	A	%96.49	**%98.78**	%97.54	%96.86	%96.71	%97.39	%96.78	%97.10	%96.76	%96.95
	S	0.015	**0.0039**	0.011	0.013	0.013	0.011	0.015	0.012	0.013	0.013
	R	10	**1**	2	5	9	3	8	4	7	6
Dermatology	A	%95.98	**%99.22**	%95.75	%96.71	%95.43	%95.34	%96.21	%96.30	%96.53	%95.61
	S	0.023	**0.0069**	0.025	0.021	0.021	0.021	0.019	0.023	0.022	0.020
	R	6	**1**	7	2	9	10	5	4	3	8
Thyroid	A	%94.34	**%98.45**	%93.41	%93.87	%93.87	%94.26	%94.42	%95.11	%93.41	%93.10
	S	0.034	**0.0111**	0.039	0.033	0.035	0.035	0.037	0.030	0.050	0.030
	R	3	**1**	7	6	6	5	4	2	7	8
Hepatitis	A	**%100**	**%100**	**%100**	**%100**	%99.78	%99.67	%99.78	**%100**	%99.78	%99.57
	S	**0**	**0**	**0**	**0**	0.008	0.009	0.008	**0**	0.008	0.011
	R	**1**	**1**	**1**	**1**	2	3	2	**1**	2	4
Heart	A	%95.25	**%96.57**	%94.92	%94.94	%95.28	%95.16	%94.98	%94.55	%95.35	%95.08
	S	0.010	**0.0034**	0.012	0.010	0.011	0.011	0.011	0.013	0.010	0.013
	R	4	**1**	9	8	3	5	7	10	2	6
**Friedman Avg Rank**		5.16	**1**	5.66	4.66	6.5	5.83	4.83	4.33	3.83	6
**Friedman Final Rank**		6	**1**	7	4	10	8	5	3	2	9

Based on this quantitative evidence, the Gauss/Mouse chaotic map was adopted as the fixed chaotic source in CSFOX to ensure consistency and reproducibility under unified experimental conditions. From an analytical perspective, the observed superiority of the Gauss/Mouse chaotic map can be explained by its ability to generate highly irregular yet bounded sequences that promote more adaptive transitions in the control variable r. Compared to other chaotic maps, this behavior reduces the likelihood of repetitive or cyclic search patterns and enables a more effective diversification of candidate solutions during the exploration phase. At the same time, the bounded nature of the sequence preserves the stability of exploitation, preventing excessive randomness in later iterations. This dual effect facilitates a more balanced exploration–exploitation trade-off, providing a principled explanation for the improved average performance and stability observed in the ablation results. The comparatively superior performance of the Gauss/Mouse chaotic map can be attributed to its underlying dynamical characteristics. Unlike several commonly used chaotic maps that may exhibit more regular or partially periodic behavior, the Gauss/Mouse map generates highly irregular and non-periodic sequences with a broad distribution over the interval (0,1). When employed to generate the exploration–exploitation control variable *r*, this behavior facilitates a more balanced and less predictable switching mechanism during the search process. Consequently, the algorithm benefits from enhanced search diversity in the early iterations while preserving effective solution refinement in the later stages, which is consistent with the observed improvements in stability and average performance across datasets.

To further validate the observed differences among chaotic maps, a Friedman test was performed based on the rank values presented in Table 2. The results show that the Gauss/Mouse chaotic map achieves the lowest average rank (1.00), indicating the best overall performance among all considered chaotic variants. Although several chaotic maps demonstrate competitive performance on specific datasets, the consistent ranking superiority of the Gauss/Mouse map across all datasets confirms that its advantage is systematic rather than incidental. These findings provide statistical evidence demonstrating that the superiority of the Gauss/Mouse chaotic map is systematic, thereby justifying its selection within the CSFOX framework.

This map is capable of generating diverse and well-distributed values within the interval (0,1), making it suitable for controlling stochastic decisions in population-based optimization algorithms without introducing additional control parameters. In CSFOX, the Gauss/Mouse chaotic map is used exclusively to generate the control variable *r*, which dynamically determines whether the algorithm performs exploration or exploitation at each iteration.

Importantly, all other algorithmic components, position update equations, and control parameters of the original FOX algorithm are preserved unchanged. By restricting the use of chaotic dynamics solely to the generation of *r*, CSFOX enhances the stochastic behavior of the search process while maintaining the structural simplicity, interpretability, and fairness of the original FOX framework.

**Fig. 5:**
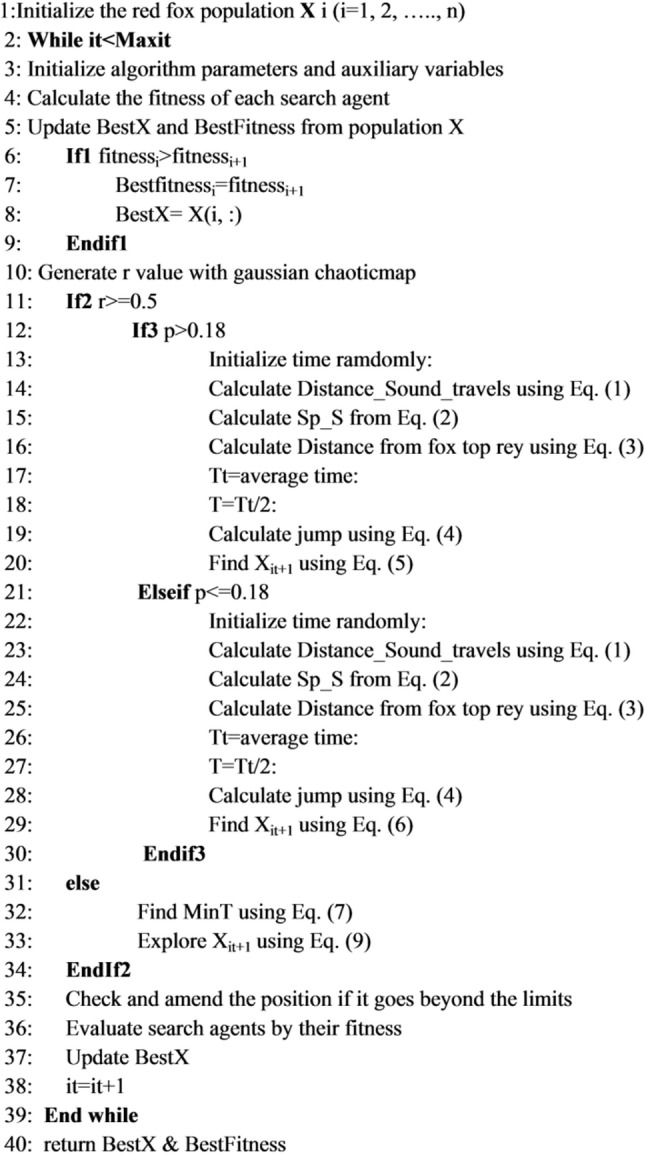
The pseudo code for chaotic fox classification algorithm (CSFOX).

The mathematical representation of the Gauss/Mouse chaotic map is provided in Eq. (10), and Fig. 5 illustrates how the chaotic variable *r* generated by this map is integrated into the FOX optimization process to regulate the balance between exploration and exploitation. The non-periodic and adaptive nature of the Gauss/Mouse chaotic sequence reduces repetitive search patterns and improves the algorithm’s ability to escape local optima. At the same time, the exploitation mechanisms inherited from FOX enable CSFOX to efficiently refine promising solutions.

Figure 5 provides a visual interpretation of how chaotic dynamics are incorporated into the FOX framework and serves as a conceptual bridge between the theoretical formulation of CSFOX and its practical implementation. Specifically, the figure presents the pseudocode of the CSFOX algorithm, summarizing the main steps of the proposed method.

In the CSFOX algorithm, the initial cluster centers are randomly initialized within the feature space to ensure diversity in the starting population. The subsequent search process then follows the original FOX update rules under the control of the chaotic exploration–exploitation switching mechanism. Figure 5 summarizes the CSFOX procedure in a step-by-step manner and clarifies how the proposed algorithm extends the original FOX framework.

The overall workflow in Lines 1–9 follows the standard FOX algorithm, including population initialization, fitness evaluation, and identification of the current best solution (***BestX***) and its corresponding fitness value (*BestFitness*). The principal distinction between FOX and CSFOX is introduced at Line 10, where the exploration–exploitation control variable *r* is generated using the Gauss/Mouse chaotic map instead of a conventional uniformly distributed random number generator. At each iteration, the chaotic value of *r* determines the subsequent search behavior.

When *r* selects exploitation (Lines 11–30), CSFOX executes the same leap-based refinement strategy as FOX. In this branch, the stochastic variable *p* governs whether the position update is performed using Eq. (5) or Eq. (6), while all distance, time, and jump computations remain identical to those of the original FOX algorithm. Conversely, when *r* selects exploration (Lines 31–34), CSFOX again follows the original FOX formulation by computing the minimum transition time *Min_T* (Line 32, Eq. (7)) and updating positions using the exploration rule defined in Eq. (9).

After either exploitation or exploration, boundary conditions are enforced (Line 35), fitness values are re-evaluated, and the global best solution is updated (Lines 36–38). This iterative process continues until the termination criterion is satisfied (Lines 39–40). In summary, CSFOX differs from FOX only in the source of stochasticity used to generate the control variable *r*. This strictly controlled modification distinguishes CSFOX from conventional chaos-enhanced algorithms, where multiple components are typically altered, and ensures that the observed performance improvements can be directly attributed to the chaotic regulation of the control variable *r*. By employing a Gauss/Mouse chaotic sequence to regulate the exploration–exploitation switching, CSFOX enhances the non-periodic and ergodic behavior of the search process while noteably preserving all original FOX operators, update equations, and parameter settings.

Preliminary experiments conducted with alternative chaotic maps did not yield practically significant improvements in classification accuracy. Therefore, the Gauss/Mouse chaotic map was adopted as the fixed chaotic source across all datasets to ensure consistency, simplicity, and reproducibility of the reported results.

For clarity, a concise comparison between the original FOX algorithm and the proposed CSFOX framework is provided in Table 3. This table summarizes how the exploration and exploitation mechanisms are preserved from FOX, while the stochastic control of the exploration–exploitation balance is enhanced in CSFOX through the chaotic generation of the control variable *r*.

**Table 3:** Comparison of exploration and exploitation mechanisms in FOX and CSFOX.

Aspect	FOX	CSFOX
Exploration–exploitation control	Random variable *r* generated uniformly	Random variable *r* generated via Gauss/Mouse chaotic map
Exploration strategy	Random walk guided by the global best solution	Same random walk, guided by chaotic sequence–driven *r*
Exploitation mechanism	Leap-based position update with fixed thresholds	Same leap-based update rules inherited from FOX
Control parameters	Fixed parameters	Same fixed parameters (no additional tuning)
Source of stochasticity	Pseudorandom number generator	Gauss/Mouse chaotic map
Algorithmic structure	Original FOX framework	FOX framework with chaotic generation of *r*

## Classification with the chaotic fox classification algorithm (CSFOX)

To ensure fairness, simplicity, and reproducibility, all control parameters were fixed across all experiments rather than being optimized for individual datasets. This design choice was intentionally adopted to avoid dataset-specific hyperparameter tuning, which may introduce bias or lead to overly optimistic performance estimates, thereby obscuring the intrinsic classification capability of the algorithm. By maintaining identical parameter settings for all datasets, the reported results reflect the generalization ability and robustness of the proposed CSFOX method under uniform experimental conditions. While parameter tuning can potentially enhance the performance of individual methods, it often introduces dataset-specific bias and may lead to overfitting, thereby limiting the reliability of comparative evaluations. In contrast, using fixed parameter settings enables a fair, consistent, and reproducible comparison, allowing the intrinsic performance and generalization capability of the algorithms to be more accurately assessed. This strategy ensures that the reported results reflect the inherent capability of each method rather than dataset-specific tuning advantages.

The population size, maximum number of iterations, and FOX-specific coefficients (*c1* and *c2*) were adopted from widely accepted standard values reported in the literature and kept constant throughout all evaluations. During the optimization-driven classification process, the number of neighboring samples considered within each cluster was fixed to *k* = *3*, based on preliminary observations indicating stable and competitive performance with this setting. The choice of k = 3 in the nearest-neighbor stage is consistent with widely adopted practices in the literature, where small odd values are commonly preferred to avoid tie conditions and to preserve local decision sensitivity. In nearest-neighbor–based classification, smaller values of k tend to produce more flexible and locally adaptive decision boundaries, whereas larger values may introduce excessive smoothing and reduce discriminative capability, particularly in heterogeneous medical datasets.

In this context, k = 3 provides a balanced trade-off between sensitivity and stability, reflecting a commonly accepted compromise between bias and variance in KNN-based decision mechanisms. Moreover, within the proposed CSFOX framework, the classification stage operates on cluster-refined data representations, which reduces sensitivity to the exact value of k. Consequently, selecting k = 3 ensures a stable and consistent configuration under unified experimental conditions^67,68^.

As a result, the reported CSFOX outcomes reflect the intrinsic classification capability of the algorithm rather than the effects of dataset-specific parameter tuning.

To perform classification using the Chaotic Fox Classification Algorithm, an appropriate performance metric must be defined for the optimization process. In CSFOX, a clustering-based classification strategy is adopted, in which cluster centers are optimized prior to class assignment. The clustering process is guided by a distance-based objective function formulated as the sum of squared errors (SSE), which is minimized during the optimization of cluster centers. Specifically, the FOX algorithm seeks to minimize the total intra-cluster squared distance among training samples, thereby promoting compact and well-separated cluster structures before assigning class labels. The objective (fitness) function used to optimize the cluster centers is defined as follows:

15 $$\min \,F = \sum\limits_{k = 1}^{K} {} \sum\limits_{{x_{i} \in C_{k} }}^{{}} {} \left\| {x_{i} - c_{k} } \right\|^{2}$$

Here, *K* denotes the number of clusters and, in all experiments, *K* was set equal to the number of classes in the corresponding dataset for both binary and multiclass cases. Accordingly, the clustering structure directly reflects the class distribution without introducing additional clusters. *x*_*i*_ represents the *i*-th training sample assigned to cluster *C*_*k*_, and *c*_*k*_ denotes the corresponding cluster center. The classification performance metrics including accuracy, sensitivity, specificity, and F1-score are evaluated after the optimization process and are not directly used as fitness values during the search. All distance computations employed for cluster assignment and nearest-neighbor voting are based on the Euclidean distance metric, ensuring consistency with the sum of squared errors (SSE) objective function used during cluster center optimization.

Although the optimization objective defined in Eq. (15) is based on the SSE criterion and does not directly incorporate class labels, this design is intentionally adopted within the CSFOX framework. The primary role of the SSE objective is to organize the data into compact and well-separated clusters, thereby capturing the underlying structure of the dataset. Rather than directly performing classification through clustering, the proposed method employs a two-stage strategy in which clustering serves as a representation learning step, followed by a supervised classification stage. Specifically, class labels are assigned using a k-nearest neighbor (k = 3) majority voting mechanism applied to the training samples. This hybrid design allows the model to benefit from both unsupervised structure discovery and supervised decision making, ensuring that the final classification outcome is guided by label information while maintaining robust and well-formed data partitions.

Prior to classification, all datasets were normalized using the Min–Max scaling method. Subsequently, 80% of each dataset was allocated for training, while the remaining 20% was reserved for testing. Using randomly initialized parameters, the training data undergo clustering based on the Chaotic Fox Classification Algorithm to identify the most suitable cluster centers, thereby completing the training phase. After training, distances between each test sample and the optimized cluster centers are computed, and cluster membership is determined accordingly.

The initial cluster centers in CSFOX are deliberately generated randomly within the normalized feature space, in accordance with standard practices in population-based metaheuristic optimization. These randomly generated centers serve solely as starting points for the training phase and do not directly determine the final classification outcomes. During training, the FOX optimization process iteratively refines the cluster centers through exploration and exploitation mechanisms until a single best-performing solution is obtained. Once this optimal set of cluster centers is identified, it is fixed and subsequently used for classification, and no further optimization or re-clustering is performed for individual test samples. As a result, classification decisions are made deterministically based on the learned cluster structure rather than repeated stochastic trials, and variations in initial cluster center positions do not constitute a decisive factor in the final classification stage.

To further mitigate any residual sensitivity to initialization, all experiments were conducted over 30 independent runs, and the reported results represent averaged statistical performance, ensuring robustness and reproducibility. Within the cluster assigned to each test sample, the classes of the nearest neighbors up to the specified number of neighbors are examined, and the sample is assigned to the most frequent class among them. In this study, the number of neighbors is fixed to k = 3. In the proposed CSFOX framework, each candidate solution encodes K cluster centers, where K corresponds to the number of classes. During the training phase, samples are assigned to these clusters based on proximity, and the clustering structure is optimized using the SSE objective function in an unsupervised manner. However, cluster assignments are not directly used as class labels. Instead, the final classification decision is determined using a k-nearest neighbor (k = 3) majority voting mechanism over the training samples. Therefore, the classification outcome depends on the local neighborhood structure rather than the global clustering configuration. This design effectively mitigates potential cluster–class misalignment, as even if clusters contain samples from multiple classes, the final label is assigned based on the dominant class among the nearest neighbors. Therefore, cluster membership serves only as a local search space restriction, while the final class decision is determined independently through nearest-neighbor voting.

The use of clustering followed by distance-based nearest-neighbor labeling is a deliberate methodological choice rather than a substitute for direct supervised classifiers. In medical diagnosis problems, class boundaries are often overlapping, nonlinear, and sensitive to noise, which may limit the robustness of purely discriminative models trained directly on labeled samples. By first optimizing representative cluster centers in a label-independent manner, CSFOX captures the intrinsic structure of the data distribution prior to class assignment. The subsequent nearest-neighbor–based labeling step enables flexible local decision making without introducing additional model complexity or strong parametric assumptions. This two-stage strategy enhances robustness, stability, and reproducibility, which are particularly important in medical data analysis.

**Fig. 6:**
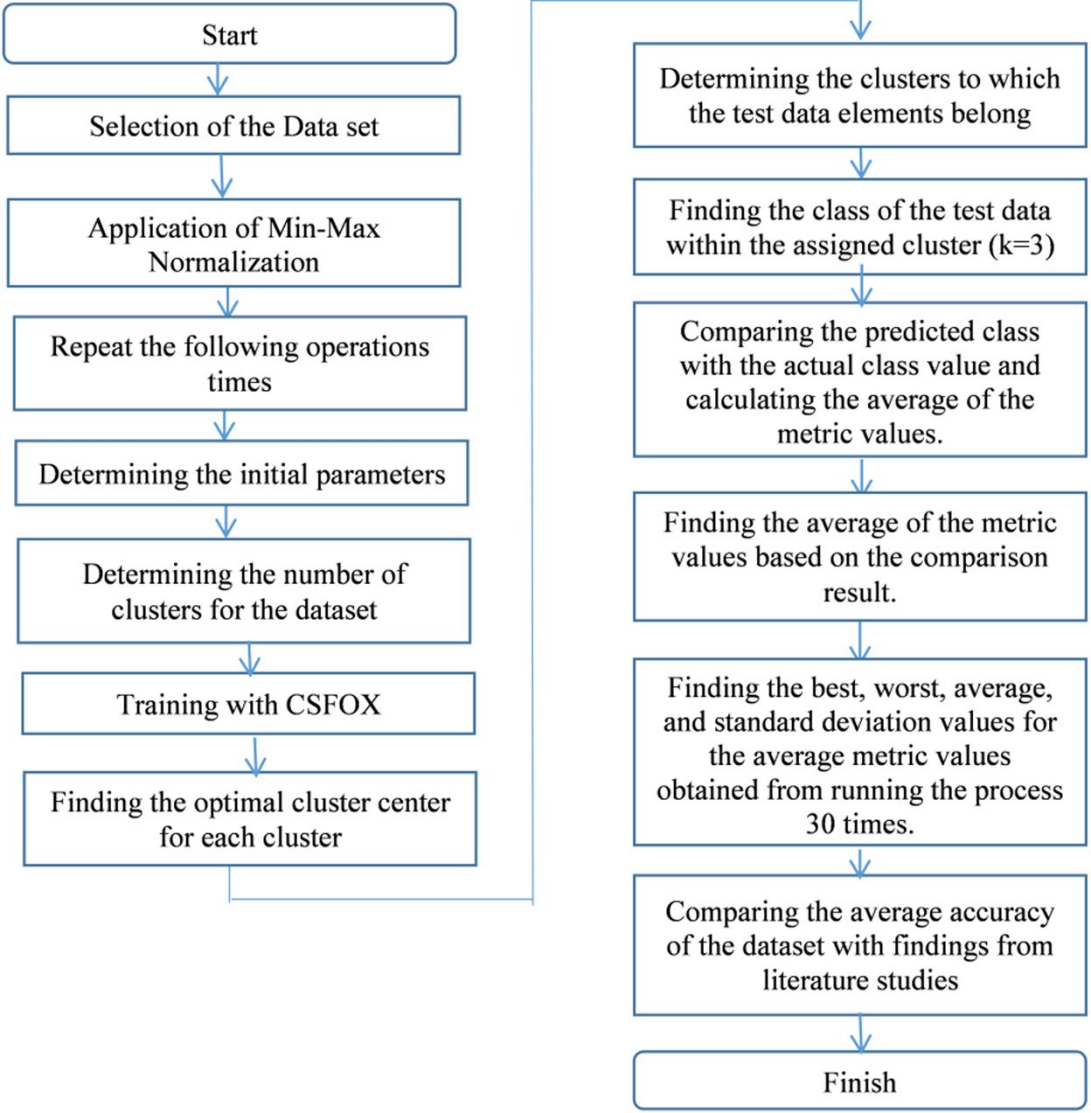
Application chart of the chaotic fox classification algorithm (CSFOX) for the classification problem.

The method was executed for 30 independent runs, and for each run, accuracy, sensitivity, specificity, and F1-score were recorded. Summary statistics, including mean, median, variance, standard deviation, best, and worst values, were computed to provide a comprehensive performance assessment. The average accuracy results obtained for each dataset were compared with those reported in similar studies using the same benchmark datasets. Figure 6 presents a flowchart illustrating how the Chaotic Fox Classification Algorithm is adapted to the classification problem, providing an intuitive overview of the complete pipeline from data preprocessing to final class assignment.

As illustrated in Fig. 6, the procedural steps applied during the classification process are summarized as follows:

- The datasets used in the study are identified.
- All datasets are scaled using the Min–Max normalization method.
- Each dataset is partitioned such that 80% of the samples are used for training and the remaining 20% for testing.
- The entire classification procedure is executed over 30 independent runs.
- Initial algorithmic parameters are defined.
- The number of clusters is set equal to the number of classes in the dataset for both binary and multiclass cases, ensuring a direct correspondence between clusters and class labels throughout all experiments.
- The algorithm is trained using the samples allocated to the training set.
- The optimal cluster centers for each cluster are identified through the CSFOX optimization process.
- Each test sample is assigned to a cluster based on its Euclidean distance to the optimized cluster centers.
- Within the assigned cluster, the class labels of the nearest neighbors determined by the predefined neighbor count are examined, and the test sample is assigned to the most frequently occurring class among them. In this study, the number of neighbors is fixed to *k* = 3*.* From a clinical interpretability perspective, the proposed classification mechanism provides a transparent and intuitive decision pathway. For a new unseen sample, the classification decision is first guided by identifying the nearest optimized cluster center, which represents a prototypical structure learned from the training data. This step offers an interpretable reference indicating which group of similar patient profiles the sample most closely resembles. Subsequently, the final class label is determined by majority voting among the nearest neighboring samples within the assigned cluster. As a result, classification decisions are not based solely on abstract optimization outcomes, but are instead grounded in proximity to representative patient groups and their observed clinical labels. Consequently, the quality of the optimized cluster centers plays a critical role in reliable class assignment, as these centers directly influence both cluster membership determination and the subsequent nearest-neighbor–based decision-making process.
- For each of the 30 independent runs, accuracy, sensitivity, specificity, and F1-score values are computed, and their mean, median, variance, standard deviation, best, and worst values are recorded.
- The average accuracy obtained for each dataset is compared with the results reported in similar studies in the literature using the same benchmark datasets.

## Experimental results and analysis

Six medical datasets were used to evaluate the effectiveness of the CSFOX algorithm for the classification problem, and the obtained results were compared with those reported in the literature using the same benchmark datasets. These datasets were selected based on their relevance to different medical conditions, enabling an assessment of the algorithm’s performance across a diverse range of disease classification tasks. Detailed descriptions of the datasets are provided in Section "Dataset", while the parameter settings used to run the proposed classification algorithm are summarized in Table 4.

**Table 4:** CSFOX classification algorithm execution parameters.

Algorithm	Parameter	Value
CSFOX	Maximum Iteration Count	1000
	Population Count	100
	Dataset Training Ratio	%80
	Dataset Test Ratio	%20
	Number of Algorithm Runs	30
	C1 and C2 Value (Fox’s Jump Movement)	0.18–0.82

The algorithm was implemented in MATLAB R2022b and executed on a computer equipped with an Intel® Core™ i5-4200 CPU @ 1.6 GHz, 4 GB RAM, and Windows 10 (64-bit) Professional operating system. To assess the effectiveness of the proposed method, the performance metrics defined in Section "Evaluation criteria" namely accuracy, sensitivity, specificity, and F1-score were employed. The source code of the proposed CSFOX algorithm will be made publicly available to ensure transparency and reproducibility.

For all datasets, 80% of the samples were used for training, while the remaining 20% were reserved for testing. The population size was fixed at 100 individuals, and all experiments were conducted over 30 independent runs to ensure statistical reliability. In each independent run, the training and testing subsets were randomly re-partitioned according to the 80%–20% hold-out scheme to ensure variability in data sampling and to provide a more robust evaluation of the proposed method. Although an explicit stratified partitioning strategy was not imposed, the use of repeated random runs, together with the additional cross-validation analysis, reduces the potential bias associated with any single split. Initially, the control parameter *a* was set to 2 and decreased linearly over the iterations. To regulate the balance between exploration and exploitation, the stochastic control variable *r* was generated using the Gauss/Mouse chaotic map. The classification accuracy values obtained in this study were compared with those reported in existing studies conducted on the same benchmark datasets.

To ensure a consistent and transparent evaluation protocol, hold-out validation was adopted as the primary performance assessment strategy for all datasets, using a fixed 80%–20% training testing split. In addition, tenfold cross-validation was applied across all datasets to further examine performance stability and generalization behavior under repeated data partitioning. These cross-validation results are reported as complementary analyses and are not intended to replace the primary hold-out evaluation. This strategy preserves methodological consistency while providing additional insight into robustness and generalization.

### Evaluation of the CSFOX algorithm

The developed CSFOX algorithm was evaluated on six different medical datasets, and the average values of accuracy, sensitivity, specificity, and F1-score obtained from 30 independent runs were reported.

#### Evaluation of CSFOX on the dermatology dataset

The CSFOX classification algorithm was evaluated on the Dermatology dataset using two complementary validation strategies, namely an 80/20 hold-out split and tenfold cross-validation. For both validation schemes, the algorithm was executed over 30 independent runs to account for the stochastic nature of the optimization process, and comprehensive statistical measures were reported. The corresponding results are summarized in Table 5.

**Table 5:** Results of the CSFOX method on the dermatology dataset.

Dataset	Validation strategy	Metric	Accuracy	Sensitivity	Specificity	F1-score
Dermatology	Hold-out (80/20)	Mean	%99.22	%99.34	%99.85	%99.21
		Median	0.9863	0.9925	0.9974	0.9880
		Variance	4.766e-05	3.697e-05	1.669e-06	4.978e-05
		Standard Deviation	0.00690	0.00608	0.00129	0.00705
		Best	%100	%100	%100	%100
		Worst	%98.63	%98.61	%99.74	%98.45
	tenfold CV	Mean	%96.47	%96.39	%99.31	%96.15
		Median	0.9647	0.9638	0.9931	0.9614
		Variance	0.00000	7.652e-08	9.635e-09	1.409e-07
		Standard Deviation	0.00000	0.00027	9.815e-05	0.00037
		Best	%96.47	%96.54	%99.36	%96.34
		Worst	%96.47	%96.38	%99.31	%96.14

As shown in Table 5, under the hold-out (80/20) validation scheme, CSFOX achieved a mean classification accuracy of 99.22%, together with high sensitivity (99.34%), specificity (99.85%), and F1-score (99.21%). The low variance and standard deviation values indicate stable and consistent performance across independent runs, while the close agreement between the mean and median values further confirms the robustness of the obtained results. Moreover, the narrow gap between the best and worst outcomes suggests limited performance fluctuation.

Under the tenfold cross-validation setting, CSFOX maintained competitive and well-balanced performance, achieving a mean accuracy of 96.47%, sensitivity of 96.39%, specificity of 99.31%, and an F1-score of 96.15%. The variance and standard deviation values observed across all evaluation metrics are numerically negligible (approximately zero) due to floating-point precision, indicating that performance variation across repeated evaluations is practically absent rather than representing meaningful statistical differences. Overall, these results demonstrate that the CSFOX algorithm delivers consistent classification performance on the Dermatology dataset under different evaluation protocols.

#### Evaluation of CSFOX on the BCWD dataset

The CSFOX classification algorithm was evaluated on the BCWD dataset using both an 80/20 hold-out validation scheme and tenfold cross-validation. To ensure statistical reliability and to account for the stochastic nature of the optimization process, all experiments were conducted over 30 independent runs. The detailed performance results obtained under both validation strategies are reported in Table 6.

**Table 6:** Results of the CSFOX algorithm on the BCWD dataset.

Dataset	Validation strategy	Metric	Accuracy	Sensitivity	Specificity	F1-score
BCWD	Hold-out (80/20)	Mean	%98.15	%96.96	%100	%97.94
		Median	0.9824	0.97.29	1	0.97.94
		Variance	2.839e-05	0.00011	0	2.917e-05
		Standard Deviation	0.00532	0.0106	0	0.00540
		Best	%99.12	%100	%100	%98.96
		Worst	%97.36	%95.45	%100	%97.05
	tenfold CV	Mean	%96.37	%95.74	%95.74	%96.10
		Median	0.9631	0.9568	0.9568	0.9601
		Variance	1.605e-06	1.849e-06	1.849e-06	1.862e-06
		Standard Deviation	0.00126	0.00135	0.00135	0.00136
		Best	%96.84	%96.16	%96.16	%96.57
		Worst	%96.31	%95.64	%95.64	%96.01

As shown in Table 6, under the hold-out (80/20) validation strategy, CSFOX achieved a mean classification accuracy of 98.15%, together with a sensitivity of 96.96%, a specificity of 100%, and an F1-score of 97.94%. The reported variance and standard deviation values are relatively low, indicating stable and consistent classification behavior across independent executions. Furthermore, the proximity between the mean and median values, as well as the limited gap between the best and worst outcomes, suggests robust performance with minimal variability.

Under the tenfold cross-validation setting, CSFOX maintained consistent and balanced performance, achieving a mean accuracy of 96.37%, sensitivity of 95.74%, specificity of 95.74%, and an F1-score of 96.10%. The small variance and standard deviation values observed across all evaluation metrics confirm the reproducibility and stability of the algorithm across different folds. Overall, these results demonstrate that CSFOX provides reliable and generalizable classification performance on the BCWD dataset under different evaluation protocols.

#### Evaluation of CSFOX on the BCWO dataset

The CSFOX classification algorithm was evaluated on the BCWO dataset using both an 80/20 hold-out validation strategy and tenfold cross-validation. To capture the stochastic behavior of the optimization process and to improve statistical reliability, all experiments were conducted over 30 independent runs. The detailed performance outcomes obtained under both evaluation protocols are summarized in Table 7.

**Table 7:** Results of the CSFOX algorithm on the BCWO dataset.

Dataset	Validation strategy	Metric	Accuracy	Sensitivity	Specificity	F1-score
BCWO	Hold-out (80/20)	Mean	%98.78	%99.57	%99.35	%98.76
		Median	0.9854	1	0.9897	0.9849
		Variance	1.592e-05	6.195e-05	2.844e-05	2.178e-05
		Standard Deviation	0.00399	0.00787	0.00533	0.00466
		Best	%100	%100	%100	%100
		Worst	%98.54	%98.03	%98.88	%98.32
	tenfold CV	Mean	%96.78	%96.37	%96.37	%96.41
		Median	0.9678	0.9637	0.9637	0.9641
		Variance	0.00000	0.00000	0.00000	0.00000
		Standard Deviation	0.00000	0.00000	0.00000	0.00000
		Best	%96.78	%96.37	%96.37	%96.41
		Worst	%96.78	%96.37	%96.37	%96.41

As reported in Table 7, under the hold-out (80/20) validation scheme, CSFOX achieved a mean classification accuracy of 98.78%, together with a sensitivity of 99.57%, a specificity of 99.35%, and an F1-score of 98.76%. The corresponding variance and standard deviation values are low, indicating stable classification behavior across independent executions. In addition, the close agreement between the mean and median values, as well as the limited difference between the best and worst results, reflects the robustness and consistency of the algorithm on the BCWO dataset.

Under the tenfold cross-validation setting, CSFOX demonstrated consistent generalization performance, achieving a mean accuracy of 96.78%, sensitivity of 96.37%, specificity of 96.37%, and an F1-score of 96.41%. The variance and standard deviation values observed across all evaluation metrics are numerically negligible (approximately zero) due to floating-point precision, indicating that performance variation across repeated evaluations is practically absent rather than representing meaningful statistical differences. Overall, these findings indicate that CSFOX delivers stable and well-balanced classification performance on the BCWO dataset under different validation strategies.

#### Evaluation of CSFOX on the thyroid dataset

The CSFOX classification algorithm was evaluated on the Thyroid dataset using both an 80/20 hold-out validation strategy and tenfold cross-validation. To account for the stochastic nature of the optimization process and to enhance statistical reliability, all experiments were conducted over 30 independent runs. The performance results obtained under both validation protocols are presented in Table 8.

**Table 8:** Results of the CSFOX algorithm on the thyroid dataset.

Dataset	Validation strategy	Metric	Accuracy	Sensitivity	Specificity	F1-score
Thyroid	Hold-out (80/20)	Mean	%98.45	%97.88	%98.75	%97.80
		Median	0.9767	0.9886	0.9907	0.9724
		Variance	0.00012	0.00042	0.00011	0.00028
		Standard Deviation	0.0111	0.0207	0.0109	0.0168
		Best	%100	%100	%100	%100
		Worst	%97.67	%94.44	%96.97	%95.22
	tenfold CV	Mean	%94.24	%89.14	%94.55	%91.12
		Median	0.9398	0.89	0.9456	0.9110
		Variance	9.523e-06	2.395e-05	4.821e-06	2.695e-05
		Standard Deviation	0.00308	0.00489	0.00219	0.00519
		Best	%94.89	%90.11	%95.03	%92.27
		Worst	%93.98	%88.77	%94.37	%90.68

As shown in Table 8, under the hold-out (80/20) validation scheme, CSFOX achieved a mean classification accuracy of 98.45%, together with a sensitivity of 97.88%, a specificity of 98.75%, and an F1-score of 97.80%. The reported variance and standard deviation values indicate stable classification behavior across independent executions. Moreover, the close correspondence between the mean and median values, along with the limited gap between the best and worst outcomes, reflects the robustness and consistency of the algorithm on the Thyroid dataset.

Under the tenfold cross-validation setting, CSFOX demonstrated reliable generalization performance, achieving a mean accuracy of 94.24%, sensitivity of 89.14%, specificity of 94.55%, and an F1-score of 91.12%. The relatively small variance and standard deviation values observed across all evaluation metrics confirm consistent performance across different folds. Overall, these results indicate that CSFOX provides stable and reproducible classification performance on the Thyroid dataset under the adopted benchmark-based validation strategies.

It is also observed that the sensitivity values under the cross-validation setting are relatively lower compared to the other performance metrics. This behavior may be attributed to class imbalance within the Thyroid dataset, where minority class samples are inherently more difficult to classify. Despite this, the overall classification performance remains well-balanced, as reflected by the relatively high specificity and F1-score values, indicating that the proposed method maintains a reasonable trade-off between correctly identifying positive cases and avoiding false positives.

#### Evaluation of CSFOX on the hepatitis dataset

The CSFOX classification algorithm was evaluated on the Hepatitis dataset using both an 80/20 hold-out validation strategy and tenfold cross-validation. To ensure statistical reliability and to reflect the stochastic nature of the optimization process, all experiments were conducted over 30 independent runs. The corresponding performance results obtained under both evaluation protocols are summarized in Table 9.

**Table 9:** Results of the CSFOX algorithm on the hepatitis dataset.

Dataset	Validation strategy	Metric	Accuracy	Sensitivity	Specificity	F1-score
Hepatitis	Hold-out (80/20)	Mean	%100	%100	%100	%100
		Median	1	1	1	1
		Variance	0	0	0	0
		Standard Deviation	0	0	0	0
		Best	%100	%100	%100	%100
		Worst	%100	%100	%100	%100
	tenfold CV	Mean	%100	%100	%100	%100
		Median	1	1	1	1
		Variance	0	0	0	0
		Standard Deviation	0	0	0	0
		Best	%100	%100	%100	%100
		Worst	%100	%100	%100	%100

As shown in Table 9, under the hold-out (80/20) validation scheme, CSFOX achieved a mean classification accuracy of 100%, together with a sensitivity of 100%, a specificity of 100%, and an F1-score of 100%. The variance and standard deviation values for all evaluation metrics are zero, indicating perfectly stable and consistent classification performance across independent executions. In addition, the equality of the mean, median, best, and worst values confirms the absence of performance fluctuation under the hold-out setting.

Similarly, under the tenfold cross-validation setting, CSFOX maintained identical performance levels, achieving 100% accuracy, sensitivity, specificity, and F1-score. The zero variance and standard deviation values observed under cross-validation further demonstrate complete reproducibility of the obtained results under the adopted evaluation protocols. While these findings indicate that CSFOX can effectively discriminate between classes in the Hepatitis dataset under the adopted experimental conditions, the relatively small sample size of the dataset should be taken into account when interpreting the results. The consistently perfect performance observed for CSFOX on the Hepatitis dataset should be interpreted with particular caution. Although all experiments were conducted under strictly controlled evaluation protocols with proper separation of training and testing samples and repeated cross-validation, the intrinsic characteristics of the Hepatitis dataset, such as its limited size and benchmark characteristics, may have inherently simplified the classification task. In addition, the underlying structure of the Hepatitis dataset may exhibit relatively well-separated or low-overlap class distributions in the feature space, which can facilitate near-perfect discrimination and contribute to the consistently identical classification outcomes observed across independent runs. Consequently, achieving 100% accuracy does not necessarily imply the absence of dataset-specific effects, nor should it be interpreted as evidence of universal superiority or real-world clinical readiness. Rather, this result reflects highly consistent performance under controlled benchmark conditions. Accordingly, further validation on larger, more heterogeneous, and independently collected clinical datasets is required to more comprehensively assess the robustness and generalization capability of the proposed method.

#### Evaluation of CSFOX on the heart dataset

The CSFOX classification algorithm was evaluated on the Heart dataset using both an 80/20 hold-out validation strategy and tenfold cross-validation. To ensure statistical reliability and to account for the stochastic nature of the optimization process, all experiments were conducted over 30 independent runs. The performance results obtained under both evaluation protocols are summarized in Table 10.

**Table 10:** Results of the CSFOX algorithm on the heart dataset.

Dataset	Validation Strategy	Metric	Accuracy	Sensitivity	Specificity	F1-score
Heart	Hold-out (80/20)	Mean	%96.57	%96.54	%96.54	%96.56
		Median	0.9656	0.9651	0.9651	0.9653
		Variance	1.203e-05	1.189e-05	1.189e-05	1.193e-05
		Standard Deviation	0.00346	0.00344	0.00344	0.00345
		Best	%97.35	%97.34	%97.34	%97.35
		Worst	%96.03	%96.03	%96.03	%96.02
	tenfold CV	Mean	%95.55	%95.50	%95.50	%95.54
		Median	0.9555	0.9550	0.9550	0.9553
		Variance	9.331e-09	8.676e-09	8.676e-09	9.317e-09
		Standard Deviation	9.66e-05	9.315e-05	9.315e-05	9.652e-05
		Best	%95.60	%95.55	%95.55	%95.59
		Worst	%95.55	%95.50	%95.50	%95.53

As reported in Table 10, under the hold-out (80/20) validation scheme, CSFOX achieved a mean classification accuracy of 96.57%, together with a sensitivity of 96.54%, a specificity of 96.54%, and an F1-score of 96.56%. The variance and standard deviation values are relatively low, indicating stable and consistent classification performance across independent executions. Moreover, the close agreement between the mean and median values, as well as the narrow gap between the best and worst results, reflects the robustness of the obtained outcomes on the Heart dataset.

Under the tenfold cross-validation setting, CSFOX maintained reliable generalization performance, achieving a mean accuracy of 95.55%, sensitivity of 95.50%, specificity of 95.50%, and an F1-score of 95.54%. The extremely small variance and standard deviation values observed across all evaluation metrics demonstrate high reproducibility and stability across folds. These findings indicate that CSFOX can effectively handle larger and more realistic medical datasets, supporting its applicability beyond small benchmark datasets and highlighting its potential for real-world clinical data analysis.

#### Convergence behavior and stability analysis

**Fig. 7:**
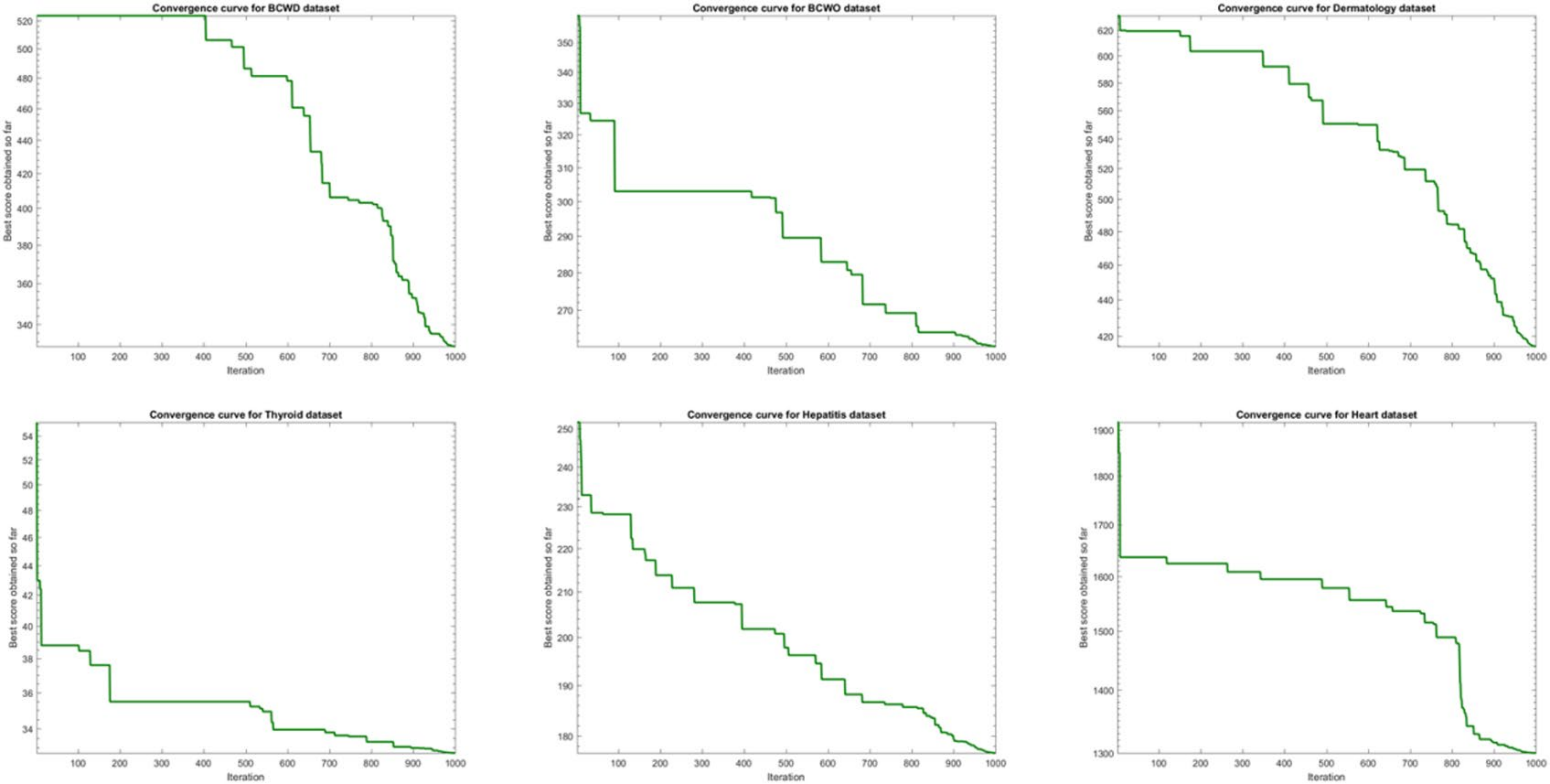
Convergence curves of the CSFOX algorithm across six medical datasets.

The convergence behavior of the proposed CSFOX algorithm across the six medical datasets is illustrated in Fig. 7, where the evolution of the best objective function value over iterations is presented to demonstrate the convergence characteristics of the algorithm. The overall convergence stability is further supported by statistical results obtained from 30 independent runs.

Figure 7 shows that CSFOX exhibits a stable and monotonic reduction in the objective function value throughout the optimization process for all datasets. Rapid performance improvements are achieved during the early iterations, indicating effective global exploration, followed by gradual refinement toward stable solutions in later stages, which suggests controlled convergence without premature stagnation. More gradual convergence patterns are observed for datasets characterized by higher heterogeneity and more complex decision boundaries, such as BCWD, BCWO, Heart, and Dermatology, while faster convergence is evident for relatively smaller or less complex datasets such as Thyroid and Hepatitis, reflecting differences in search space complexity.

In this study, convergence curves are reported based on the best-performing run in order to clearly illustrate the qualitative convergence trajectory and search dynamics of CSFOX under ideal optimization conditions. While mean convergence curves averaged over multiple independent runs can provide an overall trend, such representations may smooth out informative search behaviors and obscure distinctive convergence characteristics of population-based metaheuristic algorithms. To ensure a comprehensive assessment of stability, the average performance behavior is instead evaluated through the reported mean and standard deviation values over 30 independent runs, complemented by non-parametric statistical analyses, including the Friedman test with Kendall’s coefficient of concordance and the Nemenyi post-hoc test. This combined analysis provides a balanced evaluation of both convergence behavior and robustness without overloading the graphical presentation.

**Fig. 8:**
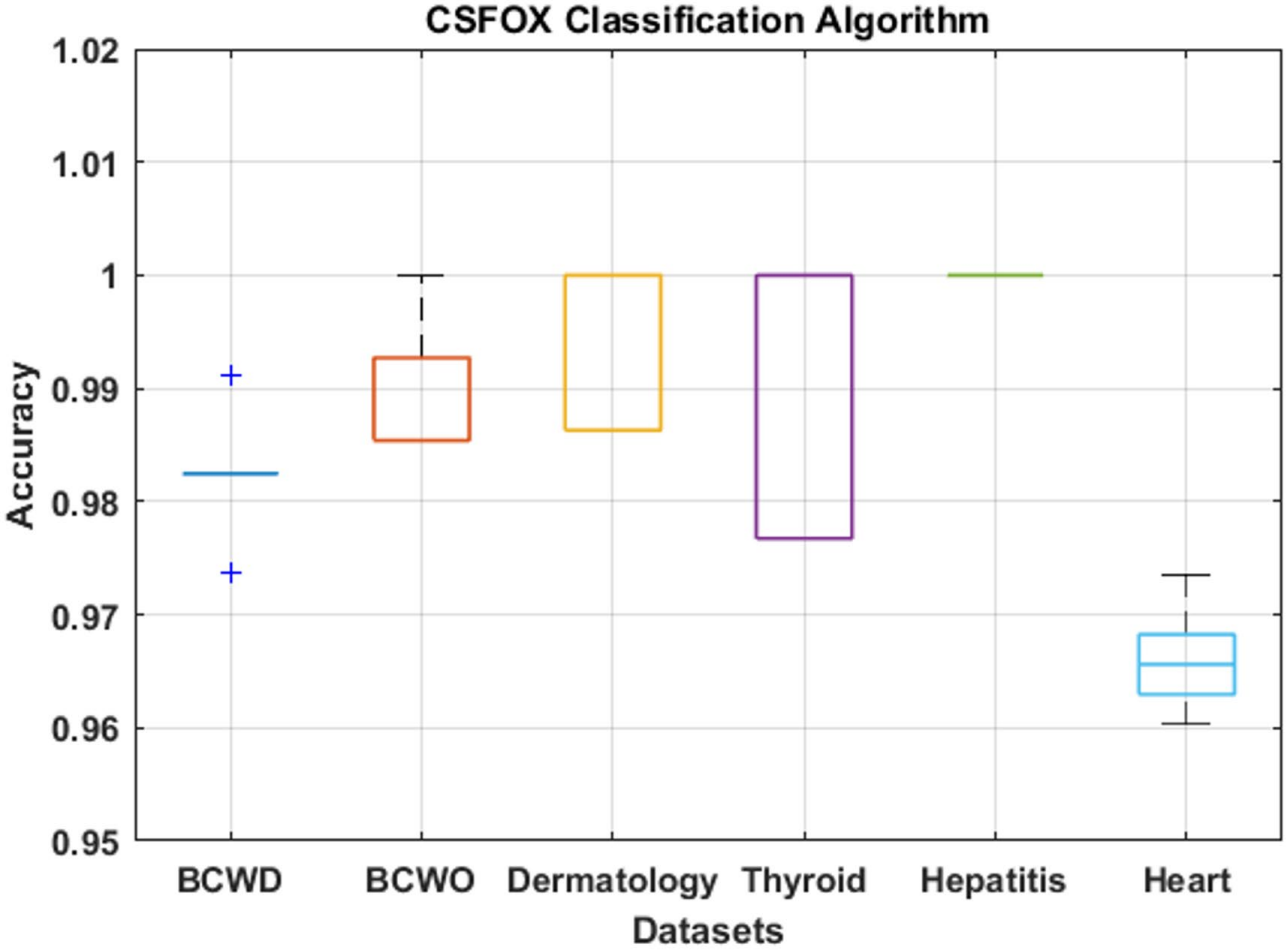
Boxplots of classification accuracy (%) obtained from 30 independent runs of the CSFOX algorithm across six medical datasets.

To further assess the stability and robustness of the proposed algorithm, the distribution of classification accuracy (%) values obtained from 30 independent runs is summarized using boxplots in Fig. 8.

Figure 8 demonstrates that CSFOX achieves consistently high classification accuracy with relatively narrow interquartile ranges across all datasets. The limited dispersion and absence of extreme outliers indicate low sensitivity to stochastic effects and confirm the robustness and repeatability of the proposed method under independent executions. These results show that CSFOX not only converges effectively toward high-quality solutions but also maintains stable performance across multiple runs, thereby providing reliable and reproducible classification outcomes.

Across the evaluated datasets, a slight decrease in performance can be observed when transitioning from the hold-out validation scheme to the tenfold cross-validation setting. This behavior may be attributed to the more rigorous and comprehensive evaluation mechanism of cross-validation, where the model is tested across multiple data partitions, thereby reducing optimistic bias associated with a single split. In contrast, the hold-out approach may yield slightly higher performance due to favorable data partitioning, particularly in relatively small or imbalanced datasets. Therefore, the observed performance differences do not necessarily indicate severe overfitting, but rather reflect the expected variation between validation strategies. Moreover, the consistent performance levels achieved across both evaluation protocols suggest that the proposed CSFOX method maintains stable generalization capability under different validation conditions.

#### ROC curve and confusion matrix analysis

**Fig. 9:**
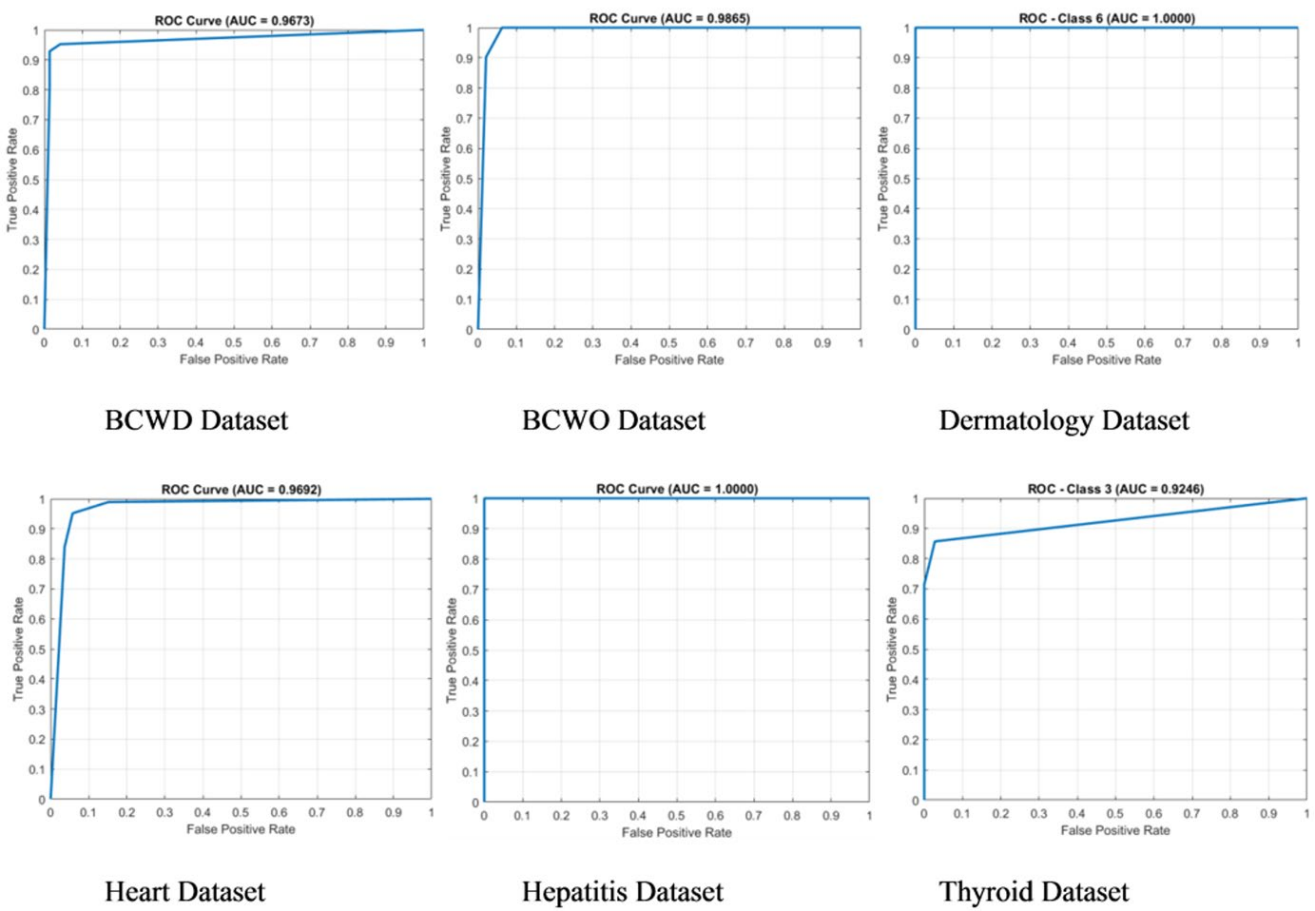
ROC curves obtained by the CSFOX algorithm for the six medical datasets based on a representative run.

To provide a deeper interpretation of the classification performance of the proposed CSFOX algorithm, Receiver Operating Characteristic (ROC) curves and confusion matrices were additionally examined for all six medical datasets. In this analysis, the presented visual results were obtained from a representative run and are intended to complement the statistical results reported in the previous sections, which are based on 30 independent runs. The ROC curves obtained for the evaluated datasets are illustrated in Fig. 9.

**Fig. 10:**
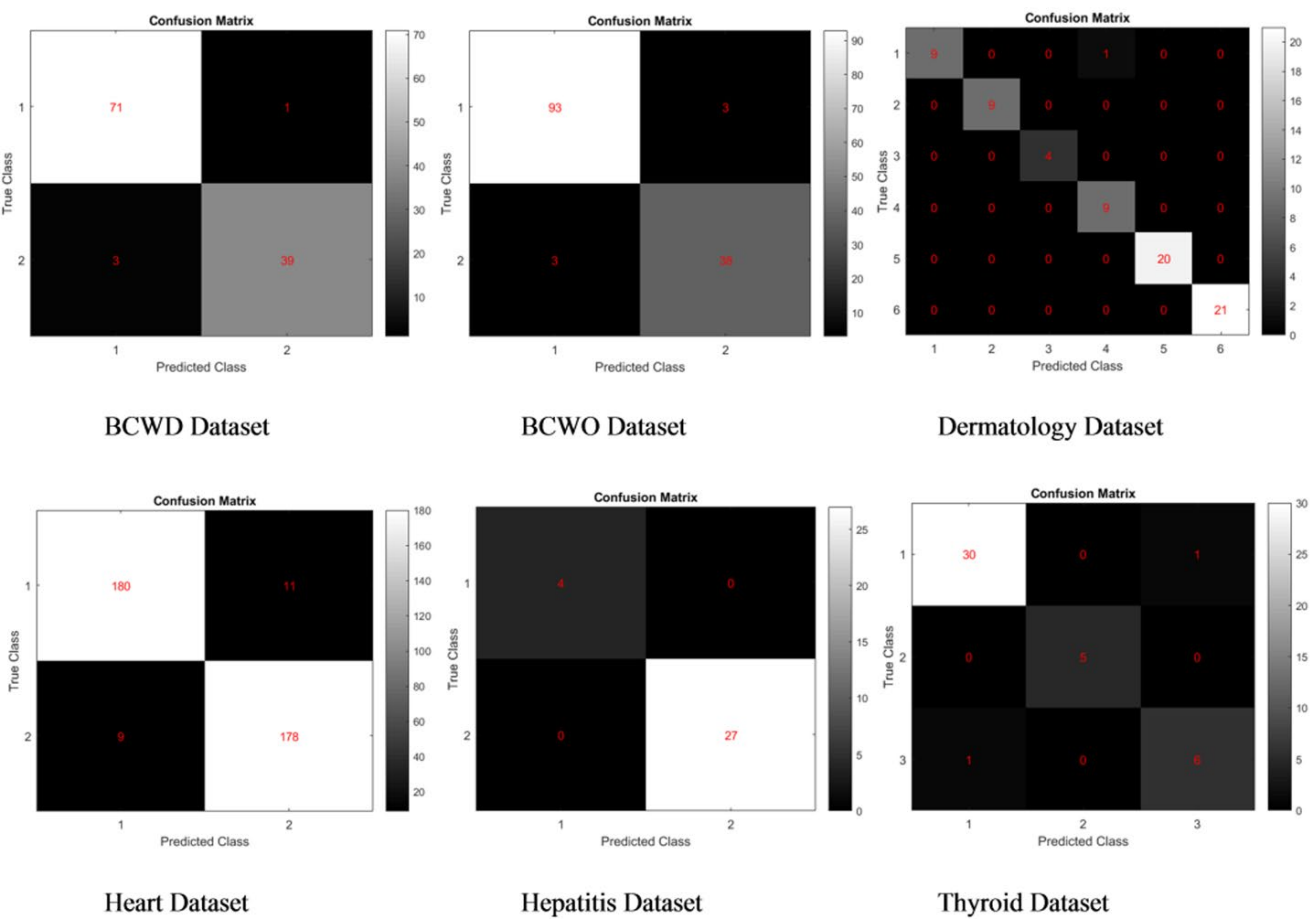
Confusion matrices of the CSFOX algorithm for the six medical datasets based on a representative run.

For the binary datasets (BCWD, BCWO, Hepatitis, and Heart), standard ROC curves are presented. For the multiclass datasets (Dermatology and Thyroid), one-vs-rest ROC curves are shown for representative classes. As shown in Fig. 9, the proposed CSFOX algorithm demonstrates strong discriminative capability across the considered medical datasets. For the binary datasets, the ROC curves are positioned close to the upper-left corner of the ROC space, indicating high true positive rates together with low false positive rates. In particular, the BCWD, BCWO, Hepatitis, and Heart datasets exhibit high AUC values, confirming the strong class-separation ability of the proposed method under representative evaluation conditions. For the multiclass datasets, the one-vs-rest ROC analyses also indicate favorable discrimination performance. The Dermatology dataset shows near-perfect class separation for the presented class-specific ROC curve, whereas the Thyroid dataset maintains a high AUC value despite its comparatively more challenging class structure. Overall, these results indicate that the proposed CSFOX algorithm preserves effective discrimination performance across both binary and multiclass medical classification tasks. In addition to ROC analysis, confusion matrices were generated to provide a class-wise interpretation of the prediction results. The confusion matrices obtained from a representative run are presented in Fig. 10.

As illustrated in Fig. 10, the confusion matrices show that the majority of samples were correctly classified across all datasets, with most predictions concentrated along the main diagonal. For the BCWD and BCWO datasets, the number of correctly classified benign and malignant samples is high, while the number of false classifications remains limited. In the Dermatology dataset, only a very small number of samples are misclassified, indicating highly reliable multiclass discrimination. The Heart dataset also presents a balanced classification structure, with relatively few errors distributed across both classes. For the Hepatitis dataset, all samples in the representative test split are classified correctly, which is consistent with the strong performance observed in the corresponding numerical results. In the Thyroid dataset, although a small number of inter-class confusions are observed, the proposed method still maintains a strong overall classification pattern with most samples correctly assigned to their respective classes. Taken together, these confusion matrices confirm that the proposed CSFOX algorithm provides stable and accurate class assignment behavior across different medical classification scenarios.

## Discussion

In this study, the Chaotic Fox Classification Algorithm (CSFOX) was introduced as a clustering-based classification framework for medical data analysis. Unlike generic chaos-enhanced metaheuristic approaches, the contribution of CSFOX lies in the purpose-driven and constrained integration of the Gauss/Mouse chaotic map to regulate the exploration–exploitation balance through the control variable *r*, while preserving all original FOX operators, update equations, and parameter settings unchanged. This design enables controlled stochastic enhancement without increasing algorithmic complexity or introducing dataset-specific tuning, thereby clearly distinguishing CSFOX from existing chaos-based classification methods.

The experimental evaluation of CSFOX was conducted on six medical datasets, including BCWD, BCWO, Dermatology, Thyroid, Hepatitis, and Heart, using both split-based and k-fold cross-validation strategies. To ensure fair and meaningful comparisons, the reported results throughout this study are organized and interpreted within their respective validation protocols, avoiding direct comparisons across different evaluation schemes. In addition, the proposed CSFOX method is evaluated under both validation strategies, enabling a consistent and balanced assessment of its performance. Furthermore, in order to ensure methodological consistency, only those studies that employ the same validation protocols (e.g., 80/20 hold-out or identical k-fold cross-validation settings) are considered for comparison. This selection strategy minimizes potential biases and enhances the fairness of the reported comparisons. The comparative results indicate that CSFOX achieves stable and competitive classification performance across the considered benchmark datasets under different validation protocols. Across all evaluated datasets, CSFOX consistently outperforms the baseline SFOX algorithm, indicating that the observed performance gains are systematic rather than dataset-specific. In particular, the improvements are consistently observable across all datasets, as demonstrated in the dataset-specific analyses. Since the only structural difference between SFOX and CSFOX lies in the chaotic generation of the control variable *r*, these improvements can be directly attributed to the enhanced exploration–exploitation balance achieved through the proposed chaotic regulation mechanism. In particular, the results obtained on the Dermatology dataset are summarized in Table 11, which illustrates the consistency of CSFOX across split-based and k-fold cross-validation settings.

**Table 11:** Contrast of presented algorithms with other studies in the field on the dermatology dataset.

Dataset	Validation strategy	Method	Accuracy rate	Final rank
Dermatology	Hold-out	YSOM^26^	%68.18	6
	Hold-out	ANFIS^69^	%95.50	4
	Hold-out	RCK^27^	%90.20	5
	Hold-out	ANN^25^	%98.36	2
	Hold-out	SFOX (Proposed Method in the Study)	%97.26	3
	Hold-out	CSFOX (Proposed Method in the Study)	**%98.63**	**1**
	K-fold CV	SVMOS^28^	%95.38	4
	K-fold CV	J48^70^	%93.50	3
	K-fold CV	EGB^71^	%95.80	2
	K-fold CV	C4.5-OAA^72^	%95.15	5
	K-fold CV	CSFOX (Proposed Method in the Study)	**%96.47**	**1**

According to the results reported in Table 11, CSFOX achieves the highest classification accuracy on the Dermatology dataset under both evaluation strategies, ranking first overall.Under the split-based evaluation setting, CSFOX attains an accuracy of 98.63%, outperforming other approaches reported in the literature. The closest competing result is obtained by the ANN-based method proposed by Al-Kahlout et al.^25^, which achieves an accuracy of 98.36%. The SFOX variant introduced in this study follows with an accuracy of 97.26%, indicating competitive performance while remaining slightly below the chaos-enhanced CSFOX framework. In the k-fold cross-validation setting, CSFOX achieves the highest classification accuracy under the k-fold cross-validation setting, securing the top final rank among the compared methods. Other k-fold-based classifiers, including SVM- and ensemble-based approaches, yield lower accuracy levels, highlighting the robustness and generalization capability of CSFOX across different validation protocols. The comparative results of the CSFOX classification algorithm on the BCWD dataset under split-based and k-fold cross-validation evaluation settings are presented in Table 12.

**Table 12:** Contrast of presented algorithms with other studies in the field on the BCWD dataset.

Dataset	Validation strategy	Method	Accuracy rate	Final rank
BCWD	Hold-out	OKNN^38^	%94.35	4
	Hold-out	FFNN^73^	%93.15	5
	Hold-out	CART^41^	%94.72	3
	Hold-out	RELIEFA^39^	%93.15	5
	Hold-out	BGWOA^40^	%94.72	3
	Hold-out	SFOX (Proposed Method in the Study)	%96.75	2
	Hold-out	CSFOX (Proposed Method in the Study)	**%98.15**	**1**
	K-fold CV	CKG^74^	%94.8	2
	K-fold CV	LGBM^75^	%93.9	3
	K-fold CV	FCM^76^	%89.2	4
	K-fold CV	CPSO^77^	%88	5
	K-fold CV	CSFOX (Proposed Method in the Study)	**%96.37**	**1**

According to the split-based evaluation results reported in Table 12, CSFOX achieves the highest classification accuracy on the BCWD dataset with a value of 98.15%, ranking first among all compared methods. The closest competing result is obtained by the SFOX variant proposed in this study, which achieves an accuracy of 96.75%, indicating that the integration of chaotic dynamics provides a clear performance gain over the non-chaotic FOX-based classifier. Other benchmark approaches, including the BGWOA method proposed by Abdel-Baset et al.^40^ and the CART-based classifier reported by Lavanya et al.^41^, yield lower accuracy levels, reflecting comparatively weaker performance under a single-split evaluation setting. In the k-fold cross-validation setting, CSFOX again secures the top final rank with a mean accuracy of 96.37%, outperforming all other compared k-fold-based methods. Competing approaches such as CKG, LGBM, and CPSO report lower accuracy values, confirming the robustness and generalization capability of CSFOX across different validation protocols. Overall, the consistent first-rank performance of CSFOX under both split-based and k-fold evaluations highlights the effectiveness of the proposed chaos-enhanced framework for breast cancer diagnosis using the BCWD dataset. The comparative results of the CSFOX classification algorithm on the BCWO dataset under split-based and k-fold cross-validation evaluation settings are presented in Table 13.

**Table 13:** Contrast of presented algorithms with other studies in the field on the BCWO dataset.

Dataset	Validation strategy	Method	Accuracy rate	Final rank
BCWO	Hold-out	LR^42^	**% 99.27**	**1**
	Hold-out	RBF + PSO^45^	% 93.55	7
	Hold-out	NNSVM^43^	% 95.38	5
	Hold-out	FEN^44^	% 96.85	4
	Hold-out	GWO	%94.67	6
	Hold-out	SFOX (Proposed Method in the Study)	% 97.61	3
	Hold-out	CSFOX (Proposed Method in the Study)	% 98.78	2
	K-fold CV	SFC^46^	% 95.57	4
	K-fold CV	BPNN^78^	%91.18	5
	K-fold CV	GSVM^79^	%96.5	2
	K-fold CV	LİBSVM^43^	%95.70	3
	K-fold CV	CSFOX (Proposed Method in the Study)	**%96.78**	**1**

According to the split-based evaluation results reported in Table 13, the logistic regression–based method proposed by Jijitha et al.^42^ achieves the highest classification accuracy on the BCWO dataset. CSFOX follows closely with an accuracy of 98.61%, demonstrating highly competitive performance relative to the best-reported split-based result. The SFOX variant proposed in this study ranks next, indicating that the incorporation of chaotic dynamics provides a consistent improvement over the non-chaotic FOX-based classifier. In the k-fold cross-validation setting, CSFOX achieves a mean classification accuracy of 96.78%, securing the top final rank among the compared methods. While certain conventional classifiers exhibit strong performance under specific split-based conditions, their performance degrades under cross-validation. The consistent first-rank performance of CSFOX under k-fold evaluation indicates stable generalization behavior across different validation protocols on the BCWO dataset.

Despite the generally strong and competitive performance of CSFOX across multiple datasets, a careful examination of the experimental results reveals several methodological and practical limitations that merit further discussion. First, the clustering-based structure of CSFOX relies on the assumption that samples belonging to the same class can be effectively represented by compact cluster centers. In datasets where class distributions exhibit significant overlap or weak cluster separability, this assumption may reduce classification effectiveness. This behavior is observed in the BCWO dataset, where the Logistic Regression method slightly outperformed CSFOX, suggesting that simpler linear decision boundaries may be more suitable for certain data characteristics.

Another aspect that warrants careful interpretation concerns the perfect accuracy results. Although CSFOX achieved 100% classification accuracy on the Hepatitis dataset, this outcome should be interpreted with caution due to the relatively small sample size and the presence of missing values. While fixed train–test splitting and mean-based imputation strategies were employed to mitigate overfitting, small datasets inherently increase the risk of optimistic performance estimation. Therefore, these results primarily demonstrate feasibility rather than definitive clinical reliability.

In addition, the use of fixed parameter settings across all datasets, while beneficial for ensuring fairness and reproducibility, may limit the adaptability of CSFOX to datasets with substantially different feature distributions or noise characteristics. Finally, due to its population-based optimization structure, CSFOX incurs a higher computational cost than conventional classifiers such as LR or SVM, which may pose scalability challenges for large-scale or high-dimensional clinical datasets without further optimization. Nevertheless, this additional computational cost is accompanied by consistent improvements in classification accuracy and stability across heterogeneous medical datasets, indicating a favorable accuracy–complexity trade-off in scenarios where predictive performance and stability are prioritized over real-time efficiency. The comparative results obtained on the Thyroid dataset under split-based and k-fold cross-validation evaluation settings are presented in Table 14.

**Table 14:** Evaluation of presented algorithms with the other studies in the field on the thyroid dataset.

Dataset	Validation strategy	Method	Accuracy rate	Final rank
Thyroid	Hold-out	SVM + RF^34^	% 93.00	3
	Hold-out	GDA_WSVMA^36^	% 91.86	5
	Hold-out	CART + RF^35^	% 86.12	7
	Hold-out	LHNFCSF^80^	%92.83	4
	Hold-out	GWO	%90.46	6
	Hold-out	SFOX (Proposed Method in the Study)	% 97.05	2
	Hold-out	CSFOX (Proposed Method in the Study)	**% 98.45**	**1**
	K-fold CV	KNN^81^	% 93.44	2
	K-fold CV	AIRSA^37^	% 85.00	5
	K-fold CV	CSFNN^82^	%91.14	3
	K-fold CV	AIRSF^37^	%85.00	5
	K-fold CV	LVQ^83^	%90.05	4
	K-fold CV	CSFOX (Proposed Method in the Study)	**%94.24**	**1**

According to the split-based evaluation results reported in Table 14, CSFOX achieves a high classification accuracy of 98.45% on the Thyroid dataset, ranking first among the compared approaches. This result indicates that the proposed chaos-enhanced framework effectively captures the underlying class characteristics of the Thyroid data. The SFOX variant proposed in this study follows CSFOX in the ranking, suggesting that the incorporation of chaotic dynamics contributes positively to classification performance, while other benchmark classifiers exhibit lower accuracy under single-split evaluation settings. In the k-fold cross-validation setting, CSFOX maintains robust generalization performance, achieving a mean classification accuracy of 94.24% and securing the top final rank among the compared methods. Although some conventional classifiers demonstrate reasonable performance under specific split-based conditions, their effectiveness decreases under cross-validation. The consistent first-rank performance of CSFOX across k-fold evaluation highlights its reliability and generalization capability for thyroid disease classification. Table 15 provides the results when CSFOX algorithm is compared with the other existing studies in the field by using the Hepatitis dataset.

**Table 15:** Evaluation of presented algorithms with the other studies in the field on the hepatitis dataset.

Dataset	Validation strategy	Method	Accuracy rate	Final rank
Hepatitis	Hold-out	LMA^32^	%94,61	5
	Hold-out	GA-MLNN^84^	%90.32	8
	Hold-out	CBR-PSO^85^	%94.58	6
	Hold-out	IG-ANFIS^86^	%95.24	3
	Hold-out	L1-SVM + ADB^87^	%89.36	9
	Hold-out	FS-F-AIRS^88^	%92.59	7
	Hold-out	GWO	%94.62	4
	Hold-out	SFOX (Proposed Method in the Study)	%96.77	2
	Hold-out	CSFOX (Proposed Method in the Study)	**%100**	**1**
	K-fold CV	GA + SVM^33^	%87,70	7
	K-fold CV	RFA^29^	%91,90	5
	K-fold CV	PSO^30^	%90,80	6
	K-fold CV	GWO	%98.04	2
	K-fold CV	RF^89^	%92.42	4
	K-fold CV	FS-AIRS^90^	%92.59	3
	K-fold CV	CSFOX (Proposed Method in the Study)	**% 100**	**1**

According to the data in Table 15, the CSFOX classification algorithm achieved a 100% classification accuracy on the Hepatitis dataset, outperforming all other methods in the literature. The second closest result to CSFOX was achieved by the second proposed SFOX classification algorithm, with an accuracy of 96.77%. The method closest to these algorithms was the LMA method presented by Mitra et al.^32^, with an accuracy of 94.61%.

These findings indicate that CSFOX demonstrates statistically supported performance on the Hepatitis dataset. Although CSFOX achieved 100% classification accuracy on the Hepatitis dataset, the relatively small size of the dataset and the presence of missing values warrant cautious interpretation of these results. To minimize the potential risk of overfitting, both split-based evaluation and k-fold cross-validation strategies were employed to ensure a fair and robust assessment of the algorithm’s performance. For handling missing values, records with incomplete critical attributes were excluded, while the remaining incomplete numerical features were imputed using the mean value strategy to maintain dataset consistency. Despite these precautions, further validation on larger, hospital-acquired datasets is required to confirm the reliability and robustness of CSFOX on Hepatitis-related predictions. To ensure the fairness of comparisons between the proposed CSFOX and SFOX algorithms and the benchmark classification methods (e.g., SVM, ANN, LR), all experiments were conducted using identical data preprocessing and dataset partitioning strategies. Specifically, the same normalization technique was applied to scale all numerical features into the range [0,1]. By applying uniform preprocessing and evaluation protocols, the observed performance differences can be attributed solely to the algorithms themselves rather than discrepancies in data handling or experimental settings. The comparative results of the CSFOX classification algorithm on the Heart dataset under split-based and k-fold cross-validation evaluation settings are presented in Table 16.

**Table 16:** Evaluation of presented algorithms with the other studies in the field on the heart dataset.

Dataset	Validation strategy	Method	Accuracy rate	Final rank
Heart	Hold-out	KNN	%94.97	4
	Hold-out	SVM	%75.02	7
	Hold-out	LDA	%74.64	8
	Hold-out	NB	%74.00	9
	Hold-out	CART	%84.86	6
	Hold-out	GWO	%93.62	5
	Hold-out	WOA	%95.11	3
	Hold-out	SFOX (Proposed Method in the Study)	%95.78	2
	Hold-out	CSFOX (Proposed Method in the Study)	**%96.57**	**1**
	K-fold CV	KNN	%95.44	2
	K-fold CV	SVM	%74.68	8
	K-fold CV	LDA	%74.94	6
	K-fold CV	NB	%73.78	7
	K-fold CV	CART	%83.95	5
	K-fold CV	GWO	%95.30	4
	K-fold CV	WOA	%95.38	3
	K-fold CV	CSFOX (Proposed Method in the Study)	**% 95.55**	**1**

According to the split-based evaluation results reported in Table 16, CSFOX achieves a classification accuracy of 96.57% on the Heart dataset, ranking among the top-performing approaches. Unlike smaller benchmark datasets, the Heart dataset represents a larger and more heterogeneous medical data structure compiled from multiple sources, providing a more realistic evaluation scenario. The strong split-based performance of CSFOX indicates its capability to effectively handle increased data complexity and variability. Under the k-fold cross-validation setting, CSFOX maintains robust generalization performance, achieving a mean classification accuracy of 95.55% and preserving a leading final rank among the compared methods. The close agreement between split-based and k-fold results suggests that the proposed algorithm does not rely on favorable data partitioning and can generalize effectively across different validation strategies. These findings demonstrate that CSFOX remains reliable when applied to larger and more diverse medical datasets, directly addressing concerns regarding scalability and real-world applicability. To provide a global statistical comparison across all evaluated datasets, the overall classification accuracy results of classical machine learning algorithms, metaheuristic-based methods (GWO and WOA), and the proposed CSFOX algorithm are summarized in Table 17.

**Table 17:** Overall comparison of classification accuracy (%) across six medical datasets.

Dataset	Metric	KNN	NB	SVM	CART	LDA	GWO	WOA	CSFOX
BCWD	Mean Accuracy	%96.37	%93.57	%97.54	%93.22	%96.23	%94.67	%96.26	**%98.15**
	Standard Deviation	0.0157	0.0323	0.0137	0.0257	0.0151	0.0091	0.0154	**0.0053**
	Rank	4	7	2	8	6	3	5	**1**
BCWO	Mean Accuracy	%96.84	%96.16	%96.98	%94.82	%96.01	%95.62	%96.79	**%98.78**
	Standard Deviation	0.0143	0.0157	0.0162	0.0220	0.0195	0.0115	0.0155	**0.0039**
	Rank	3	5	2	8	6	7	4	**1**
Dermatology	Mean Accuracy	%96.89	%80.14	%97.35	%93.52	%96.44	%94.01	%96.58	**%99.22**
	Standard Deviation	0.0176	0.0607	0.0180	0.0259	0.0209	0.0242	0.0190	**0.0069**
	Rank	3	8	2	7	5	6	4	**1**
Thyroid	Mean Accuracy	%95.74	%97.36	%90.16	%93.57	%92.71	%90.46	%95.58	**%98.45**
	Standard Deviation	0.0367	0.0264	0.0452	0.0333	0.0390	0.0341	0.0353	**0.0111**
	Rank	3	2	8	5	6	7	4	**1**
Hepatitis	Mean Accuracy	%98.92	%89.14	%100	%100	%88.60	%94.62	%98.71	**%100**
	Standard Deviation	0.0176	0.0460	0	0	0.0470	0.0258	0.0262	**0**
	Rank	2	5	**1**	**1**	6	4	3	**1**
Heart	Mean Accuracy	%94.97	%74.00	%75.02	%84.86	%74.64	%93.62	%95.11	**%96.57**
	Standard Deviation	0.0111	0.0174	0.0213	0.0182	0.0184	0.0081	0.0111	**0.0034**
	Rank	3	8	6	5	7	4	2	**1**
**Friedman Mean Rank**		3.1667	6.1667	3.6667	5.8333	6.1667	6.0000	3.8333	**1.1667**

Beyond the dataset-specific analyses, Table 17 presents a unified comparison of classical machine learning algorithms, metaheuristic-based classifiers (GWO and WOA), and the proposed CSFOX method across six medical datasets using a rank-based statistical evaluation. For consistency in the global statistical ranking analysis, the Friedman test reported in Table 17 was conducted using split-based accuracy results, while k-fold cross-validation outcomes were examined in detail within the dataset-specific comparisons. According to the mean rank results derived from the Friedman analysis, CSFOX achieves the lowest mean rank (1.16), indicating the best overall performance across all datasets. Among the baseline methods, KNN attains the second-best mean rank (3.16), demonstrating that distance-based learning remains a strong and competitive classical approach for medical classification tasks, followed by WOA as the next most competitive metaheuristic-based classifier, while GWO exhibits comparatively weaker overall ranking performance. In contrast to both classical and existing metaheuristic approaches, CSFOX consistently secures the top rank for each dataset, including cases where identical accuracy values lead to shared first positions. The statistical reliability of these observations is further confirmed by the Friedman test (*p* = 0.0012), and the corresponding Kendall’s coefficient of concordance (W = 0.56) indicates a strong effect size, demonstrating that the performance advantage of CSFOX is not only statistically significant but also practically meaningful across diverse and heterogeneous medical classification tasks. This value indicates a moderate-to-strong level of agreement among the rankings, confirming that the observed performance differences are consistent across datasets rather than being influenced by random variations. Following the Friedman analysis, a Nemenyi post-hoc test was conducted to examine pairwise performance differences among the compared methods. he critical difference (CD) value at α = 0.05 was calculated as 4.6867. The post-hoc results indicate that CSFOX exhibits statistically significant superiority over CART, GWO, and WOA, as the corresponding rank differences exceed the CD threshold, while the differences with the remaining baseline classifiers do not reach statistical significance under the conservative Nemenyi criterion.

In addition to the global comparison presented in Table 17, a more focused baseline analysis was performed to examine the behavior of the proposed CSFOX framework in relation to two widely used and interpretable reference strategies, namely standalone KNN and the hybrid K-means + KNN approach. The comparative results obtained over six medical datasets, based on 30 independent runs, are presented in Table 18.

**Table 18:** Comparative performance of CSFOX, KNN, and K-means + KNN in terms of classification accuracy across six medical datasets (30 independent runs).

Dataset	Metric	KNN	K-MEANS + KNN	CSFOX
BCWD	Mean Accuracy	%96.37	%95.49	**%98.15**
	Standard Deviation	0.0157	0.0240	**0.0053**
	Rank	2	3	**1**
BCWO	Mean Accuracy	%96.84	%96.52	**%98.78**
	Standard Deviation	0.0143	0.0140	**0.0039**
	Rank	2	3	**1**
Dermatology	Mean Accuracy	%96.89	%96.80	**%99.22**
	Standard Deviation	0.0176	0.0170	**0.0069**
	Rank	2	3	**1**
Thyroid	Mean Accuracy	%95.74	%93.33	**%98.45**
	Standard Deviation	0.0367	0.0303	**0.0111**
	Rank	2	3	**1**
Hepatitis	Mean Accuracy	%98.92	%99.57	**%100**
	Standard Deviation	0.0176	0.0140	**0**
	Rank	3	2	**1**
Heart	Mean Accuracy	%94.97	%95.03	**%96.57**
	Standard Deviation	0.0111	0.0125	**0.0034**
	Rank	3	2	**1**
**Friedman Mean Rank**		2.33	2.66	**1.0**

As shown in Table 18, the proposed CSFOX framework consistently achieves the highest mean classification accuracy across all evaluated datasets. While standalone KNN demonstrates competitive performance on certain datasets, and K-means + KNN provides a reasonable clustering-assisted baseline, both approaches generally remain below the accuracy levels obtained by CSFOX. This behavior can be attributed to the optimization-driven clustering mechanism of CSFOX, where cluster centers are adaptively refined through the FOX search process enhanced by chaotic regulation. This design enables the method to capture the underlying data structure more effectively prior to the final nearest-neighbor–based labeling step. In contrast, standalone KNN relies primarily on local distance relationships, whereas the K-means + KNN approach depends on cluster assignments generated by a deterministic centroid update process without global search capability.

In addition, the relatively low standard deviation values observed for CSFOX indicate a more stable classification behavior across independent runs. A Friedman rank analysis further supports these observations. According to the results presented in Table 18, CSFOX achieves the best overall mean rank with a value of 1.00, followed by KNN with a mean rank of 2.33 and K-means + KNN with a mean rank of 2.66. This ranking pattern suggests that the performance advantage of CSFOX is consistently maintained across different datasets rather than being limited to specific cases. These findings provide additional evidence regarding the effectiveness of the proposed CSFOX framework in comparison with standalone KNN and the K-means + KNN baseline under unified experimental conditions.

In this study, robustness is interpreted in a statistical sense, referring to the consistency and stability of the algorithm’s performance across multiple independent runs and different datasets. This aspect is evaluated through non-parametric statistical analysis, including the Friedman test and the Nemenyi post-hoc procedure, as well as Kendall’s coefficient of concordance (W), which quantifies the level of agreement among rankings. In addition, the performance results obtained from 30 independent runs further support the stability of the proposed method. Moreover, the ablation study results presented in Table 2 further support the robustness of the proposed method by demonstrating consistent performance across different chaotic-map configurations, where only the chaotic component is varied while all other algorithmic elements are kept fixed. These findings collectively indicate that the CSFOX framework produces stable and reliable results rather than performance gains limited to specific experimental conditions.

Overall, the results demonstrate that the proposed CSFOX and SFOX methods consistently achieved the best classification accuracy across nearly all datasets. On the BCWO dataset, the LR method proposed by Jijitha et al. slightly outperformed the proposed CSFOX classification algorithm. Additionally, SFOX, the other proposed algorithm, achieved performance values close to those of CSFOX, indicating competitive behavior. Taken together, these findings suggest that the proposed CSFOX method provides effective and reliable solutions for classification problems.

The superior performance of CSFOX can be attributed to the integration of chaotic dynamics, which enhances the algorithm’s ability to explore the solution space more effectively. By introducing controlled randomness, chaotic maps help prevent premature convergence and promote a more diverse search process. This mechanism enables CSFOX to adapt to datasets with varying levels of complexity, highlighting its potential as a general-purpose classifier for medical diagnosis.

It is important to clarify that the experimental results reported in this study are obtained from controlled evaluations conducted on publicly available benchmark datasets that originate from real clinical measurement processes. While these findings demonstrate the methodological effectiveness of CSFOX as a classification framework, they should not be interpreted as evidence of immediate clinical readiness or real-world deployment. Accordingly, references to potential integration into clinical decision support systems or electronic health record platforms are intended to be prospective and exploratory rather than confirmatory in nature.

Although the datasets used in this study are derived from real clinical measurements, they are publicly available benchmark datasets. Therefore, the absence of independent external validation datasets (e.g., hospital-acquired data) represents a limitation of the current study. Therefore, the contribution of this study should be interpreted as methodological rather than as direct clinical deployment.

The benchmark datasets employed in this study originate from real clinical measurement processes; therefore, they inherently include natural sources of variability and noise (e.g., measurement uncertainty, recording inconsistencies, and population heterogeneity). Accordingly, the reported results reflect performance under realistic, imperfect data conditions rather than on purely synthetic or noise-free inputs. Nevertheless, the present study does not conduct an explicit noise- or perturbation-based robustness analysis in which controlled levels of label noise, feature-level perturbations, or outlier contamination are systematically injected. Therefore, robustness claims in this work are limited to stability across independent runs and data partitioning strategies. More comprehensive noise or perturbation analyses have been considered for design and will be evaluated in subsequent or future studies.

It should also be explicitly acknowledged that fixing all control parameters across datasets represents a deliberate methodological trade-off. While this strategy enhances fairness, transparency, and reproducibility by preventing dataset-specific hyperparameter tuning, it may limit the algorithm’s ability to achieve dataset-wise optimal performance. Certain datasets may benefit from alternative population sizes, iteration counts, or neighborhood configurations that better reflect their intrinsic structure and complexity. Accordingly, the reported results primarily reflect the robustness and generalization capability of CSFOX under unified experimental conditions rather than its maximum attainable performance on individual datasets.

Accordingly, claims regarding robustness and general applicability in this study are restricted to benchmark-based experimental settings and should not be interpreted as direct evidence of clinical generalization in the absence of external or hospital-acquired validation cohorts.

Several datasets considered in this study, such as Hepatitis and Thyroid, are relatively small in size. Although controlled validation strategies were employed throughout the experimental evaluation, small-sample medical datasets may inherently increase the risk of optimistic performance bias by simplifying the underlying classification task. Consequently, very high or perfect performance values observed on such datasets should be interpreted with caution and should not be regarded as definitive evidence of universal superiority or direct clinical applicability. This limitation further underscores the necessity of validating the proposed approach on larger, more heterogeneous, and independently collected clinical datasets to reliably assess its robustness and generalization capability.

In addition, deep learning–based models were not included in the comparative analysis of this study due to the specific characteristics of the datasets and the methodological scope of the work. The datasets employed consist of small- to medium-sized tabular medical data, for which deep learning architectures typically do not offer a consistent performance advantage without substantially larger sample sizes, extensive hyperparameter tuning, or additional regularization strategies. Furthermore, deep learning models generally require complex architectural design choices and increased computational resources, which may reduce interpretability and introduce experimental bias in small-sample clinical studies. Accordingly, this study deliberately focuses on optimization-driven and classical classification methods that are better aligned with the data structure and experimental objectives considered here.

In addition, the comparative analysis in this study primarily focuses on classical classifiers and optimization-based methods, which aligns with the clustering-driven nature and experimental scope of the proposed CSFOX framework. Although modern tree-based ensemble models and deep learning architectures have demonstrated strong performance in various medical classification tasks, their effectiveness often depends on large-scale datasets, extensive hyperparameter tuning, and reduced model interpretability. Given the small- to medium-sized tabular datasets considered in this work, such comparisons were intentionally excluded to avoid introducing experimental bias and to maintain methodological consistency. Nevertheless, extending the comparative analysis to include tree-based ensembles and deep learning models represents an important direction for future research.

Beyond its classification performance, CSFOX has the potential to offer strong implications for real-world healthcare applications. Its ability to achieve high accuracy across multiple datasets suggests that it could be considered for integration into clinical decision support systems (CDSS) to assist healthcare professionals in diagnostic decision-making. Furthermore, CSFOX may be incorporated into electronic health record (EHR) systems as a supportive analytical component, enabling data-driven insights during clinical assessment. Such integration has the potential to support clinical workflows by reducing diagnostic uncertainty and facilitating early disease detection.

Although CSFOX has demonstrated promising results on publicly available medical datasets, additional validation under real clinical conditions is required. Future studies should focus on testing CSFOX with hospital-acquired datasets and evaluating its effectiveness in diagnosing specific diseases, such as cancer or neurological disorders, where classification accuracy plays a critical role in patient management. Additionally, further methodological enhancements, such as adaptive chaotic mapping strategies, could be investigated to improve the robustness and computational efficiency of CSFOX in real-time or large-scale classification scenarios.

### Computational complexity and scalability analysis

The computational complexity of the proposed CSFOX classification framework is mainly determined by three components: (i) population-based optimization, (ii) fitness evaluation during cluster center optimization, and (iii) distance-based classification of test samples. Let *N* denote the population size, *D* the feature dimension, *I* the maximum number of iterations, *C* the number of clusters (equal to the number of classes), and *M* the number of training samples. During each iteration, CSFOX updates the positions of all fox agents and evaluates their fitness values. The position update operations inherited from the FOX algorithm involve vector-wise arithmetic computations with linear complexity *O(D)* per agent. Consequently, the overall time complexity of the position update stage is *O(N* × *I* × *D)*. The fitness evaluation stage requires computing distances between candidate cluster centers and training samples, resulting in a computational cost of *O(N* × *M* × *D)* per iteration. Therefore, the total complexity of the optimization phase can be expressed as *O(I* × *N* × *(D* + *M* × *D))*, which simplifies to *O(I* × *N* × *M* × *D)* in practical scenarios where *M »1*, indicating that the number of training samples is sufficiently large.

After the optimization phase, classification of test samples is performed using a distance-based assignment mechanism. For each test instance, distances to the optimized cluster centers are computed with a complexity of *O(C* × *D)*, followed by a local k-nearest neighbor analysis within the assigned cluster. Since both *C* and *k* are typically small constants, this stage introduces negligible computational overhead compared to the optimization process. The incorporation of chaotic maps in CSFOX does not impose additional computational burden, as chaotic number generation involves constant-time arithmetic operations. This is because chaotic number generation relies on simple deterministic mathematical mappings involving basic arithmetic operations (e.g., multiplication and modulus), which have computational complexity comparable to conventional pseudo-random number generation. Therefore, the use of chaotic sequences does not introduce any additional overhead beyond standard random number generation mechanisms. Consequently, the asymptotic time complexity of CSFOX remains equivalent to that of the original FOX-based classification framework. From a scalability perspective, CSFOX is well suited for medium-scale medical datasets commonly used in experimental clinical decision support studies. Although population-based optimization methods inherently incur higher computational costs than conventional classifiers such as Logistic Regression or Support Vector Machines, the optimization phase of CSFOX is executed offline during model training. Once the optimal cluster centers are obtained, the online inference stage relies solely on lightweight distance computations, rendering CSFOX suitable for real-time or near-real-time diagnostic applications.

Moreover, the algorithm is inherently parallelizable across population members and fitness evaluations, allowing efficient acceleration on multi-core CPU architectures or GPU-based platforms. These characteristics support the scalability of CSFOX for larger datasets and practical deployment scenarios.

In addition to runtime considerations, the suitability of CSFOX for large-scale or high-dimensional medical datasets should be interpreted in light of two practical factors. First, because the optimization phase includes repeated distance evaluations, the training-time cost increases with both the number of samples (*M*) and the feature dimension (*D*), consistent with the derived complexity term *O(I* × *N* × *M* × *D)*. Second, the current implementation relies on Euclidean distance within an SSE-driven objective, which may become less discriminative as dimensionality increases due to the well-known concentration of distances in high-dimensional spaces. Therefore, although CSFOX is effective for small- to medium-sized tabular medical datasets under the adopted experimental setting, direct application to very high-dimensional feature spaces (e.g., omics data) or very large cohorts may require additional strategies such as dimensionality reduction or feature selection, alternative distance metrics or metric learning, and scalability-oriented training protocols (e.g., sampling/mini-batching, early stopping, or distributed/parallel fitness evaluation). These considerations represent an important limitation of the present study and motivate future work toward large-scale and high-dimensional clinical learning scenarios.

## Conclusion

This study introduced the Chaotic Fox Classification Algorithm (CSFOX), a chaos-enhanced extension of the FOX optimization framework designed for medical data classification. The experimental findings indicate that incorporating chaotic dynamics into the FOX search mechanism improves optimization behavior and yields competitive classification performance under controlled benchmark evaluation settings.

The results demonstrate that chaos-based regulation of the exploration–exploitation balance can serve as an effective strategy for enhancing optimization-driven classifiers without increasing algorithmic complexity. From a methodological perspective, CSFOX illustrates how chaotic control can be systematically integrated into population-based optimization algorithms to improve classification stability and consistency under unified experimental conditions.

While the reported findings are promising, the present study is limited to publicly available benchmark datasets. Accordingly, further validation using hospital-acquired data, robustness-oriented evaluation protocols, and real-time implementation studies is required before considering practical clinical deployment. Future research will also investigate adaptive chaotic strategies and hybrid classification schemes to further enhance scalability and generalization behavior. In particular, dynamic chaotic map selection mechanisms that adaptively switch among different maps based on convergence behavior or population diversity indicators represent a promising research direction.

The transition of the proposed framework toward clinical deployment will be addressed in future studies through a series of concrete steps. These include validation on hospital-acquired datasets collected under routine clinical conditions, collaboration with clinical experts to ensure clinically meaningful feature interpretation, and the integration of data preprocessing pipelines compliant with real-world electronic health record standards. In addition, hybrid supervised extensions of CSFOX will be explored by incorporating label-guided learning mechanisms, where optimized cluster centers are combined with supervised classifiers such as support vector machines or neural networks to enhance predictive performance while preserving interpretability.

Overall, this work provides a solid experimental foundation for future investigations into chaos-based optimization frameworks for medical data classification and related clinical decision support research.

## Data Availability

All datasets used in this study are available at: [http://archive.ics.uci.edu/ml](http:/archive.ics.uci.edu/ml).
